# Coherence Potentials: Loss-Less, All-or-None Network Events in the Cortex

**DOI:** 10.1371/journal.pbio.1000278

**Published:** 2010-01-12

**Authors:** Tara C. Thiagarajan, Mikhail A. Lebedev, Miguel A. Nicolelis, Dietmar Plenz

**Affiliations:** 1 Section on Critical Brain Dynamics, National Institute of Mental Health, Bethesda, Maryland, United States of America; 2 Department of Neurobiology, Center for Neuroengineering, Duke University, Durham, North Carolina, United States of America

## Abstract

Transient associations among neurons are thought to underlie memory and behavior. However, little is known about how such associations occur or how they can be identified. Here we recorded ongoing local field potential (LFP) activity at multiple sites within the cortex of awake monkeys and organotypic cultures of cortex. We show that when the composite activity of a local neuronal group exceeds a threshold, its activity pattern, as reflected in the LFP, occurs without distortion at other cortex sites via fast synaptic transmission. These large-amplitude LFPs, which we call *coherence potentials*, extend up to hundreds of milliseconds and mark periods of loss-less spread of temporal and amplitude information much like action potentials at the single-cell level. However, coherence potentials have an additional degree of freedom in the diversity of their waveforms, which provides a high-dimensional parameter for encoding information and allows identification of particular associations. Such nonlinear behavior is analogous to the spread of ideas and behaviors in social networks.

## Author Summary

Perception and behavior are thought to arise from transient associations among sub-groups of nerve cells in the brain. However, identifying which of the many active neurons are associated at any given time and how poses a challenge. Here we show that when the composite activity of a local group of cortical neurons, measured as a complex waveform in the extracellular field, exceeds a threshold, its activity pattern extending up to hundreds of milliseconds occurs without distortion at other cortical sites via fast synaptic transmission. We call these all-or-none propagated patterns “coherence potentials”, in analogy to action potentials at the single cell level. In contrast to action potentials, which are stereotypical and thus capable only of binary coding, coherence potentials are diverse and complex waveforms that can serve as a high-dimensional parameter for encoding information. The non-linear relationship between local activity and its extent of replicated spread suggests a “tipping point” that bears analogy to the propagation of innovations and economic behavior in social networks, which can spread rapidly once they have garnered a local critical mass.

## Introduction

Since its introduction by Hebb [1], the transient formation of cell assemblies has been one of the most fundamental and provocative hypotheses to understand cortex function. The existence and identification of such assemblies, however, has been extremely difficult as it requires a criterion that separates the activities of neurons inside the cell assembly from those outside the assembly. Assuming that neurons within an assembly undergo similar changes in their activities, spectral coherence in the local field potential (LFP) reflecting temporal similarities irrespective of amplitudes [2] and synchronization in the spiking activity of individual neurons [3]–[6] have each been used independently to identify functionally associated neuronal groups at different cortical sites during discrete responses to stimuli (for review see [7]–[9]). Many studies focused particularly on similarities in the LFP, as temporal attributes of the LFP waveform have been shown to carry substantial information about a stimulus or behavior [10]–[12], often serving as better predictors of behaviors than spikes [13]–[15]. For instance, in the primate motor and premotor cortex transient phase-locking across cortical sites can be seen in the β- or γ- frequency band of the LFP during voluntary movements and behavioral task performances [16]–[19], which has been proposed to reflect neuronal interactions [20]. In the EEG, transient phase-locking correlates with “gestalt” perception clearly linking similarities in phase across cortical sites to higher cortical function [21]. Importantly, spontaneous or ongoing activity shares many similarities with stimulus-evoked activity [22]–[26], and indeed, transient phase-locking has been observed during ongoing cortical activity in awake monkeys [10],[23]. Because transient phase-locking has been found to include different cortical sites at different times, these dynamics have been widely appreciated in the literature under the concept of “metastability” [8],[27]–[34], emphasizing the idea that cortical dynamics might be sequentially ordered as strings of transient associations.

If similarity is the defining property of associative activity, then the exact *replication* of activity at different sites might be the ultimate degree in similarity that can be utilized by the cortex. Such precise replication of activities requires the transient phase-locking over a wide range of frequencies in addition to maintaining the amplitude of the signal. Therefore, we analyzed the similarity of the complete LFP waveform across cortical sites during spontaneous cortical activity. Ongoing activity was recorded from chronically implanted arrays in the motor, premotor, and somatosensory regions of two monkeys sitting quietly in a recording chair as well as from organotypic cultures from rat cortex. We demonstrate a sigmoidal relationship between the amplitude of a deflection in the spontaneous LFP, reflecting the aggregate neuronal activity at a single cortical site and the occurrence of the identical LFP waveform *and* amplitude at other sites with millisecond delays. We show that this phenomenon of all or none, loss-less propagation of activity at high amplitudes arises by virtue of synaptic transmission. These results suggest a “tipping point” in the cortical network dynamics analogous to that found for the spread of ideas, innovation, and economic behavior in social networks, where a small increase in the number of participating agents can suddenly lead to widespread cascades of adoption.

## Results

### Waveform Correlations across Sites Increase Non-Linearly with Amplitude

Ongoing LFP activity (∼40 min) was recorded from two macaque monkeys sitting in a monkey chair without having to attend to stimuli or perform motor commands. An array of thirty-two microelectrodes spanning 64 mm^2^ was implanted in the left motor cortex (M1_left_) and one array of sixteen electrodes spanning ∼34 mm^2^ was implanted in the left somatosensory cortex (S1_left_) in monkey A. Four arrays of sixteen electrodes spanning 34 mm^2^ were located in the left and right dorsal premotor and motor cortex areas, respectively, of monkey B (see [35] for a sketch of the in vivo array positions and electrode configurations). For the in vitro analysis, spontaneous LFP activity was recorded from organotypic prefrontal and somatosensory cortex cultures of rat grown for many weeks on sixty channel planar integrated microelectrode arrays spanning 2 mm^2^ [36].

**Figure 1:**
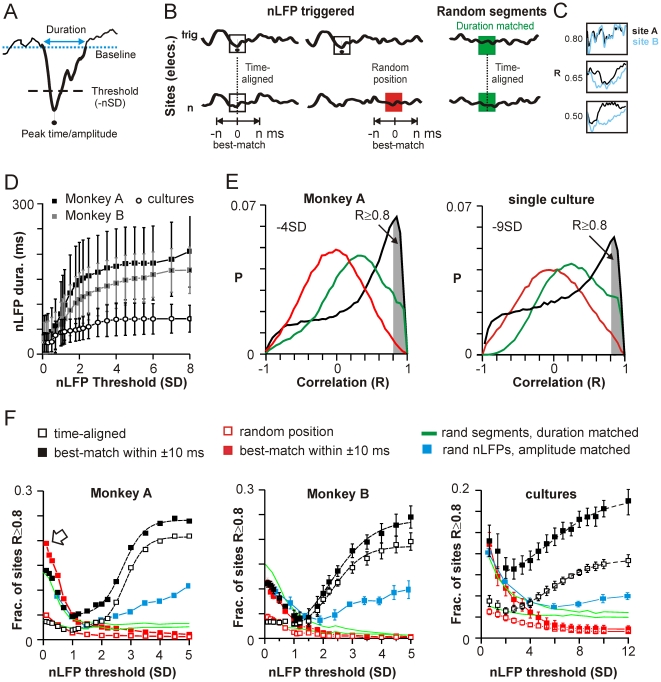
Increasing nLFP amplitude is associated with a sigmoidal transition to a regime of widespread waveform similarity. (A) The nLFP is defined as the complete negative excursion (*thick line*) from baseline or average activity (*dotted line*). Significant nLFPs are identified if they cross a negative threshold of *n* times the standard deviation (−n SD) estimated from the total time course of the recording. Amplitude and time of the nLFP correspond to the negative peak. (B) Schematic of nLFP triggered comparisons to time-aligned and best-match (up to ±*n* ms shifted in time) periods at each other electrode (*left*). As controls, nLFP triggered periods were compared to random periods of similar duration (*middle*). A priori similarities of LFP segments were estimated from random, time-aligned segments, whose duration distribution was matched to that of nLFP segments (*right*; see also D). Color code extends to (D) and (E). (C) Waveforms were compared by calculating the correlation *R* of the time series. Examples of simultaneous waveforms at two sites A and B with *R* = 0.5, 0.65, and 0.8. (D) Mean duration ±SD of the nLFP as a function of nLFP threshold (M1_left_; monkey A; average over all four arrays in monkey B; average of *n* = 6 cultures). (E) Distributions of correlation *R* (average across all electrodes) between nLFP triggered and time-aligned (*black*), nLFP triggered and random position (*red*), and random and time-aligned (*green*) periods as described in (B) shows a large fraction of correlated sites for nLFP triggers with threshold −4 SD in vivo (*left*; M1_left_; monkey A) and −9 SD in vitro (*right*; single culture). (F) The average fraction of highly correlated sites across events (i.e., *R*≥0.8, shaded area in Figure 1E) increases sigmoidally with nLFP amplitude beyond ∼1 SD (*broken line*; sigmoidal fit, *R*>0.99 all cases) and was significantly different from all controls (random, duration- and amplitude-matched; *p*<10^−4^ all cases). Best-match comparisons (shown here up to a maximum shift of ±10 ms) demonstrate higher correlations overall (*open arrow*: best-match comparison artificially increases the likelihood of finding highly correlated, short segments at low threshold within ±10 ms). *Left*: M1_left_; monkey A. *Middle*: average over all four arrays in monkey B. *Right*: average of *n* = 6 cultures.

The LFP has been shown to carry substantial information about the underlying spike activity [14],[37],[38]. In our datasets in vivo, amplitudes of negative deflections in the LFP (nLFPs) correlated with both the rate of spike firing as well as the number of distinct units, indicating that the nLFP amplitude reflects the firing rate and degree of synchronization in the local neuronal population [35]. Similarly, the nLFP strongly correlates with the time of neuronal spiking in the in vitro cultures [39]. We thus defined our periods of interest as continuous negative excursions from the baseline (i.e., nLFPs; Figure 1A) whose peaks exceeded thresholds defined in terms of successive multiples of the standard deviation of the signal (SD). These nLFPs varied substantially in duration. Mean duration increased with thresholds up to −1.5 SD and then changed little, if at all, for nLFPs with higher amplitudes reaching about 170 ms in monkeys A and B and 60 ms in vitro (Figure 1D).

For each amplitude threshold, we randomly selected up to 250 suprathreshold nLFPs on each channel (per threshold 4,000–8,000 nLFPs in vivo, and 10,000–15,000 nLFPs in vitro). For each of these nLFPs (we call them triggers), we compared its waveform to the identical period recorded at each other electrode (*time-aligned*, Figure 1B) by calculating the correlation coefficient *R* of the time series, which provides a measure of the temporal similarity of the waveform independent of amplitude (examples in Figure 1C and Figure S1). As controls, we calculated similarities between trigger nLFPs and randomly selected duration-matched periods at other electrodes (Figure 1B, *red*) as well as similarities between random, time-aligned segments, whose durations distributed similarly to the trigger nLFPs (Figure 1B, *green*; see also Material and Methods “Correlation Analysis and Controls”). At high nLFP detection thresholds, many of the time-aligned nLFPs were highly correlated. The distribution of these correlations showed a characteristic peak close to 0.9 that was not present in the controls (Figure 1E; representative distributions for M1_left_ in monkey A at −4 SD and a single culture at −9 SD). We thus systematically evaluated the average fraction of sites that were correlated *R*≥0.8 for time-aligned comparisons (Figure 1E, *gray area*) as we increased the amplitude threshold from low to high SD (Figure 1F). Remarkably, when the nLFP amplitude exceeded approximately −1.5 SD, there was a rapid transition to a large fraction of correlated sites in the time-aligned comparison that was absent in the controls (Figure 1F, *open squares*; *p*<10^−4^ for ≤−2 SD, both controls; Kolmogorov-Smirnov (KS) test). This demonstrates an amplitude dependent, sigmoidal transition from a regime of low spatial coherence to one of high spatial coherence (sigmoidal fit *R*>0.99, *broken line*; χ2/DoF<10^−4^ all cases compared to *R* values between 0.86 and 0.91 for linear fits). This transition was also found for the somatosensory cortex S1_left_ in monkey A (Figure S2A), suggesting that, in line with the in vitro data, the transition is not limited to motor cortex. Note that we use the term coherence here to refer to waveform similarity in a general sense rather than the more technical spectral coherence, which is limited to a particular frequency band.

### Correlated nLFPs Are Temporally Clustered with Millisecond Delays

In order to take into account potential time delays between trigger nLFPs and similar waveforms at other electrodes, we repeated our analysis, allowing the window of comparison (whose size was determined by the duration of the trigger nLFP) to shift by up to ±10 ms relative to the trigger nLFP (Figure 1B) in order to identify the temporal shift that gave rise to the highest correlation (*best-match*). The sigmoidal transition to a state of high spatial coherence was robust to this best-match comparison (Figure 1F, *filled black squares*). Importantly, the significant increase in the fraction of correlated sites for best-match comparisons beyond −1.5 SD suggested that a number of highly correlated nLFPs were shifted in time. We note that the fraction of sites with highly correlated waveforms also increased as the threshold was lowered from −1.0 SD towards 0 SD particularly for best-match comparisons (Figure 1F, *open arrow* in left panel), which simply reflects the increased likelihood of finding correlations for shorter and shorter segments within a relatively larger time window (cf., Figure 1D).

**Figure 2:**
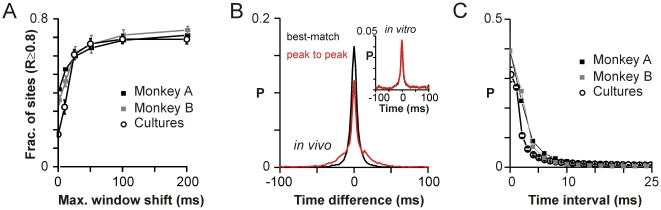
Correlated waveforms are temporally clustered. (A) Correlated periods with *R*≥0.8 occur within ±50 ms. Fraction of sites with best-match correlation *R*≥0.8 plateaus beyond ±50 ms as maximum shift is increased for up to ±200 ms (nLFP threshold of −4 SD in vivo and −9 SD in vitro; M1_left_, monkey A; average over four arrays in monkey B; average over *n* = 6 cultures). (B) The distribution of time differences between the trigger nLFP and highly correlated nLFPs at other electrodes is centered at 0. Time-shifts corresponding to best-match periods (*red*) correspond closely to the time difference between the peaks of the nLFP (*peak-to-peak*; *black*; M1_left_ from monkey A). *Inset*: representative culture. (C) Highly correlated nLFPs occur within quick succession, less than 10 ms apart from each other. Density distribution of time intervals between successively correlated sites identified using nLFP triggers of −4 SD in vivo and −9 SD in vitro on any given electrode (M1_left_, monkey A; average over four arrays in monkey B; average over *n* = 6 cultures).

A systematic analysis of time shifts up to ±200 ms relative to the time-aligned position revealed that the fraction of highly correlated sites increased up to a maximal shift of ±50 ms but changed little thereafter for both monkeys and in vitro (Figure 2A). Thus, most correlated periods at other electrodes occurred within ±50 ms of a trigger nLFP. Note that the maximal average fraction of correlated sites as a function of trigger amplitude is achieved when there are about 30%–40% available sites on the array, and thus the upper part of the sigmoidal function cannot be explained by a ceiling effect due to limited array size. The average distribution of time differences between the trigger nLFP and highly correlated nLFPs at other sites centered around 0, suggesting that any site on the array can be preceded or followed by highly correlated events. The distribution was similar for pairs of electrodes, indicating that the time differences arose from functional rather than anatomical factors such as differences in cortical depth (Figure S3). As expected, the distribution in temporal shifts corresponding to best-match comparisons closely matched the peak-to-peak nLFP intervals (Figure 2B, Figure S2B).

When highly correlated nLFPs identified as above were arranged according to their position in time, the distribution of inter-event intervals revealed that similar nLFPs arose on the array in quick succession with delays rarely exceeding 10 ms (Figure 2C; see also Methods “Time-Shift Analysis” for calculation of delays). In fact, the distribution was skewed heavily towards zero; i.e., many similar nLFPs peaked in the same time bin. We note that such simultaneous occurrence of nLFPs does not necessarily exclude a propagation process as an underlying mechanism (see below). As demonstrated in Figure S4, the spread between highly correlated nLFPs often entailed a one-to-many cascade rather than a strict one-to-one sequence resulting in many zero-delay nLFPs within a cascade. In addition, propagation could have occurred faster than the temporal resolutions of our recordings, which were 2 ms in vivo and 1 ms in vitro. Time delays ranging between 1 and 50 ms translate to propagation speeds of 0.02–1 m/s in vivo and 0.004–0.2 m/s in vitro given inter-electrode distances of 1 mm and 0.2 mm, respectively. This range is generally consistent with stimulus evoked spike response latencies in the organotypic slice [40] as well as stimulus evoked wave initiation in the acute slice [41].

### Correlated Waveforms Are Highly Similar in Amplitude

**Figure 3:**
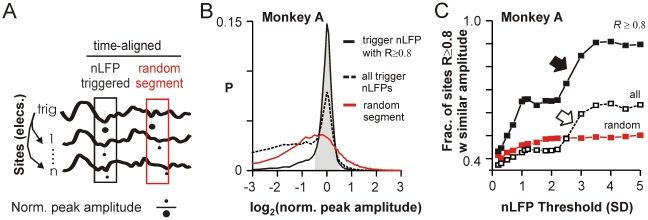
Correlated waveforms are similar in amplitude. (A) Schematic of amplitude comparison where the peak amplitude within the time-aligned window at each site was normalized by the peak amplitude of the trigger nLFP (elec_amp_/nLFPtrig_amp_). Comparison of time-aligned random segments was used as a control (*red*). (B) Distributions of log-normalized values as in (A) for correlated waveforms with peaks ≤−4 SD from the M1_left_ array in monkey A distribute narrowly around 0 (i.e., log 1). Inclusion of waveforms at all sites irrespective of correlation broadens the original distribution towards ratios <1. The sharp peak at 0 is absent in random segment comparisons. (C) Fraction of sites with peak amplitude within ±50% of the nLFP trigger amplitude (shaded area in B) increased non-linearly as a function of trigger amplitude and was significantly greater for correlated waveforms alone (see Figure S4 for monkey B and cultures).

We next performed a similar analysis for amplitude, comparing the peak amplitude of the trigger nLFP to the largest negative peak within the time-aligned window of comparison at other sites (Figure 3A). When we restricted this analysis only to sites with correlated waveforms (*R*≥0.8), we found that the normalized peak amplitude values distributed narrowly around 1 (Figure 3B; M1_left_ monkey A; shown in log scale; 0 = log_2_(1)), indicating that the amplitudes of correlated waveforms were highly similar. When the analysis was extended to include all sites, irrespective of the correlation of their waveforms, the distribution retained its peak around 1, but now broadened because of the inclusion of many amplitude ratios <1 originating from non-similar waveforms with on average smaller amplitudes than the trigger nLFP. As a further control, time-aligned comparisons of random segments with durations comparable to those of trigger nLFPs lacked a sharp peak at 1 demonstrating a low a priori probability of waveforms having similar peak amplitudes. Importantly, the fraction of sites with similar peak amplitudes (i.e., within ±50%, Figure 3B shaded area) increased non-linearly as a function of nLFP amplitude threshold in parallel to the increase in waveform similarity (Figure 3C, *open arrow*; data for monkey B and in vitro shown in Figure S5). This transition was equally pronounced when only highly correlated waveforms were considered (*R*≥0.8; Figure 3C, *filled arrow*), and indeed, amplitude similarity was greater for the more correlated waveforms. These results indicate a non-linear transition to a regime where there is not only high similarity in waveform at different sites but amplitude as well.

Finally, we demonstrate that the sigmoidal transition to a spatially extended regime of high coherence was not due to an overall greater similarity of nLFPs with large amplitudes, as one might expect due to an improved signal-to-noise ratio. We estimated the a priori likelihood of finding similar nLFP waveforms at distant times as a function of amplitude. We compared nLFP triggers at each threshold to nLFPs with similar amplitude at each other electrode randomly chosen from time periods at least 200 ms away from the peak of the nLFP trigger. The fraction of such comparisons that were highly correlated (*R*≥0.8) was 2- to 3-fold smaller even for the highest amplitudes both in vivo and in vitro (Figure 1E, *blue*; *R* = 0.12±0.01 to 0.27±0.01 for amplitudes ≤−4 SD in vivo; 0.52±0.01 to 0.6±0.01 for amplitudes ≤−9 SD in vitro). This implies that while large amplitude nLFPs clustered in time were highly similar, temporally distant clusters were highly dissimilar.

### Visualization of Coherence Potential Sequences

**Figure 4:**
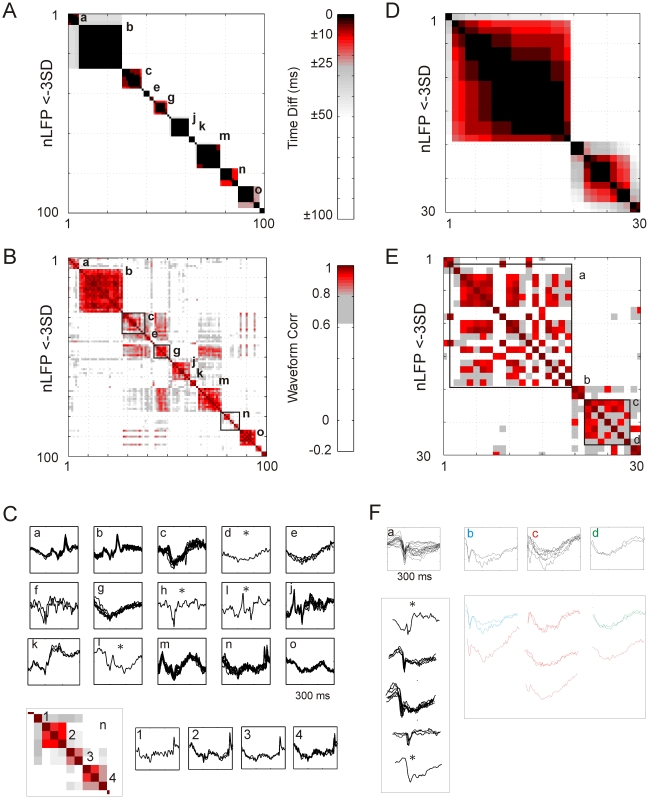
Visualization of coherence potential sequences. (A) Matrix of time delays for 100 suprathreshold nLFPs (≤−3 SD) that occurred in temporal succession on the array in M1_Left_ of monkey A. Most nLFPs formed tight temporal clusters (“a”, “b”, …, “o”; *black*) that were well separated from successive clusters (i.e., >50 ms, *white*). Clusters are potential candidates for coherence potentials. The color coding for time delays was based on the range of delays between highly correlated nLFPs (*R*≥0.8; see Figure 2B) where ∼95% were <10 ms (*black*), ∼4% were between 10 and 25 ms (*black*–*red*), a fraction of a percent were between 25 and 50 ms (*red*–*gray*), and virtually none were >150 ms (*white*). (B) Corresponding matrix of waveform correlations. nLFP waveforms within clusters (max. delay of 10 ms) tend to be highly similar (*black*–*red*; *R*≥0.8), i.e. coherence potentials. nLFPs between clusters (i.e., successive coherence potentials) were no more similar than random nLFPs (*white*). Color code for waveform similarity was based on the distribution of correlations for nLFP triggered and random time-aligned comparisons (cf., Figure 1E). *Red*: *R*≥0.8, correlations roughly around the peak in Figure 1E. *Gray–white*: 0.6<*R*<0.8, correlations between the peak of nLFP triggered comparisons and the intersection with random comparisons. *White*: *R*≤0.6, not distinguishable from random. (C) Over-plots of waveforms corresponding to clusters “a” through “o”. A coherence potential is composed of suprathreshold nLFPs from at least two sites on the array that are highly similar in waveform and amplitude. Note the similarity of nLFP waveforms within a coherence potential and the variability between successive coherence potentials. *Asterisk*: Single waveforms that fail the operational definition of a coherence potential, which requires at least two sites. *Below*: Details of cluster “n”. This cluster is characterized by waveforms with a relatively short-duration nLFP. Consequently, best-matched nLFP comparison only incompletely captures the similarity of the waveforms over the longer period of ∼300 ms. (D) An example of a time matrix for thirty successive suprathreshold nLFPs (≤−3 SD) in the same monkey later in time relative to (A) which shows less compact temporal clustering. (E) Corresponding correlations of nLFP waveforms from panel (D) reveal a “checkerboard” pattern within temporal clusters indicating an intermingling of a small number of distinct waveforms, i.e. temporally overlapping but spatially separated coherence potentials. (F) Over-plot of waveforms from temporal clusters “a” to “d” from panel (D). Although over-plots based on a simple temporal criterion (i.e., maximum delay of 10 ms) did not result in identical waveforms, sorting the waveforms based on a correlation threshold criterion (here *R*≥0.8) readily uncovers the multiple coherence potentials that were temporally intermingled. Thus, temporal cluster “a” is composed of five distinct waveform groups of which three were identified as coherence potentials, while temporal cluster “c” consisted of five distinct waveforms of which one grouped with cluster “b” and three with cluster “d” (color code indicates regrouping; *asterisk*: waveforms that fail the operational definition of a coherence potential).

We have thus far shown that, as the nLFP amplitude increases, there is an abrupt transition to a regime where a pattern of activity appears at a large number of sites with millisecond delays without distortion of its temporal structure or substantial change in amplitude. We will call these highly similar temporally clustered nLFP waveforms with similar large amplitude “coherence potentials” to reflect their wide spatial coherence. Given that the extent of spatial coherence can vary considerably, we operationally define a coherence potential as an nLFP waveform with suprathreshold amplitude (right side of the sigmoidal function) that occurs at least at one other site in the network. In Figure 4, we provide a detailed visualization of individual coherence potentials as they are identified in the successive occurrence of 100 suprathreshold nLFPs (i.e., ≤−3 SD) during a segment of recording from M1_left_ in monkey A (note that no information on the spatial positions of the nLFPs is used in this representation). First, we plotted in matrix form the time difference between all nLFP peaks (Figure 4A), which reveals temporal clusters of nLFPs (i.e., within a few milliseconds of one another, *black–red* squares along the diagonal). The corresponding correlation matrix (Figure 4B, see also Material and Methods “Visualization of Coherence Potentials”), which measures the similarity of nLFP waveforms to each other, reveals that temporally clustered suprathreshold waveforms (separated by <10 ms) tend to be highly similar in waveform (*red*). In contrast, waveforms of successive temporal clusters (generally separated by >50 ms, *white*) were no more similar to one another than randomly chosen nLFPs. Thus, in most cases, coherence potentials are readily identified in the ongoing activity by simply over-plotting successive suprathreshold nLFPs arising with a maximum delay of 10 ms from each other (note that single waveforms, by definition, cannot classify as coherence potentials and are marked by an asterisk). These over-plots readily demonstrate the similarity of waveforms and their amplitudes within a coherence potential, and the diversity of waveforms across coherence potentials (Figure 4C).

In other instances, the temporal clusters of suprathreshold nLFPs were less precisely delimited (Figure 4D; e.g., clusters *a* and *c*) reflecting temporally intermingled coherence potentials that gave rise to a “checker board”–like organization in the correlation matrix (Figure 4E). Here, although over-plots based on a simple temporal criterion (i.e., maximum delay of 10 ms) did not result in identical waveforms, sorting the waveforms based on a correlation threshold criterion (here *R*≥0.8) readily uncovered the multiple coherence potentials that were temporally intermingled (Figure 4F). Thus, temporal cluster *a* was composed of five distinct waveform groups of which three were coherence potentials, while temporal cluster *c* consisted of five distinct waveforms of which one grouped with cluster *b* and three with cluster *d* (see Figure S6 for more raw coherence potential traces in vivo and in vitro).

### Coherence Potentials Are Spectrally Diverse, Negative–Positive Waveforms

**Figure 5:**
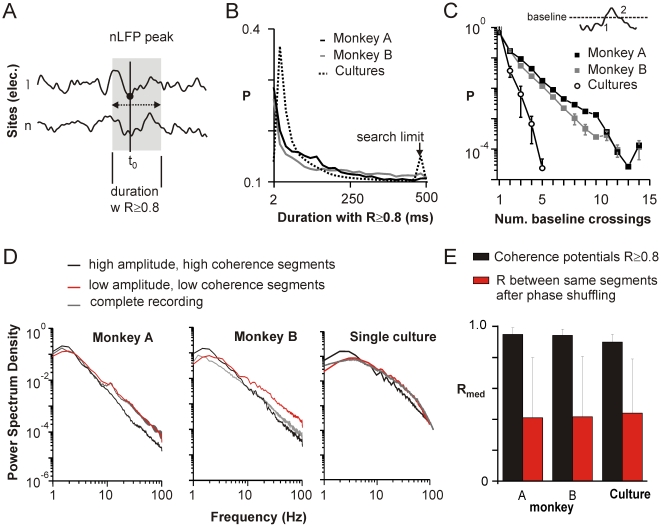
Coherence potentials are spectrally diverse negative–positive waveforms. (A) Sketch showing method of identifying periods around the nLFP for which sites maintained correlation of *R*≥0.8. Starting from the peak of an nLFP identified on a given electrode (≤−4 SD in vivo, ≤−9 SD in vitro) the period of time-aligned comparison (*gray*) was increased in each direction (*arrows*) up to ±500 ms or until *R* dropped below 0.8. Duration was taken as the combined period in both directions. (B) Durations distributed with a heavy tail spanning a wide range up to 500 ms with median values of 200 ms and 178±22 ms in monkey A (M1_left_) and monkey B (average of four arrays), respectively, and 92±12 ms in vitro (*n* = 6 cultures). (C) Corresponding distribution of the number of negative excursions over the duration of the correlations. Roughly 90% included only one baseline crossing indicating that correlated periods generally included part of one negative and one positive excursion. In the remaining fraction, periods with *R*≥0.8 extended over multiple negative excursions with exponentially decreasing likelihood. This demonstrates that the nLFP periods capture a large fraction of the full extent of the correlation around negative peaks validating our initial approach that focused on nLFPs. (D) Power spectrum density for correlated periods both in vivo (*left*, *middle*) and in vitro (*right*) shows a systematic decay with higher frequencies (*black*), similar to the power spectrum for non-correlated periods (*red*) and the complete recording (*grey*). (E) Coherence potentials involve specific phase relationships among frequencies. Median correlation (R_med_±SD) for coherence potentials (*R*≥0.8) after phase shuffling of their waveforms shows dramatic loss of correlation.

We then studied the waveform characteristics of coherence potentials in more detail and asked in a first step whether the negative excursion of the nLFP described their full extent in time (cf., Figure 4A–C; clusters “a”, “b”, “n”). For each suprathreshold nLFP (≤−4 SD in vivo; ≤−9 SD in vitro), we systematically increased the time of comparison before and after the nLFP peak for up to ±500 ms (Figure 5A) or until *R*<0.8. The identified durations with *R*≥0.8 distributed with a heavy tail spanning a wide range up to 500 ms with median values of 200 ms (Monkey A, M1_left_) and 178±22 ms (monkey B, average of four arrays), respectively, and 92±12 ms in vitro (*n* = 6 cultures). Thus, coherence potentials can last for up to many hundred milliseconds. During this period, most coherence potentials included a combination of one negative and one positive excursion from the baseline (>90%; Figure 5C). Indeed, hardly 10% of coherence potentials extended over more than three baseline crossings, the minimal expected number for a two-cycle oscillation, suggesting that they did not originate from an underlying oscillation.

Significantly, the period of highest correlation was initiated at a fast rise phase generally around the −1 SD mark but continued for up to several hundreds of milliseconds after the last threshold crossing (Figure S7). Thus, while the nLFP alone does not describe the full extent of the correlation, it is a sufficient approximation for the purpose of this study and provides a useful method of extracting periods of interest.

The power spectrum density (PSD) of coherence potentials rarely revealed dominant oscillations. We calculated the PSD for coherence potentials whose negative component (i.e., nLFP) was at least 256 ms long, using exactly 256 ms for the power spectrum analysis. The shortness of these segments restricts the reliable region of the PSD to frequencies >4 Hz for which the PSD showed a systematic decay without any dominant frequency band in the aggregate (Figure 5D) as well as at the level of individual coherence potentials. A similar decay was observed for periods of equal durations uncorrelated with other sites (i.e., −0.3≥*R*≤0.3, *red*) and the complete recording (Figure 5D). In order to get a more accurate view of frequencies below 4 Hz, longer segments of ±4 s around the trigger nLFP were also considered, but again the decay was devoid of dominant frequencies (see Figure S8A, S8B for examples of individual segments). Thus, while coherence potentials do not reflect a specific oscillatory period, this does not exclude the possibility that some coherence potentials could have oscillatory components. Indeed, autocorrelation analysis revealed oscillatory components at various frequencies in a small fraction of individual coherence potentials (Figure S8C). Importantly, when coherence potentials were phase-shuffled, the correlations between sites were destroyed (Figure 5E), indicating that coherence potentials involve specific phase relationships among frequencies.

### Coherence Potential Sequences Arise by Virtue of Fast Synaptic Transmission

**Figure 6:**
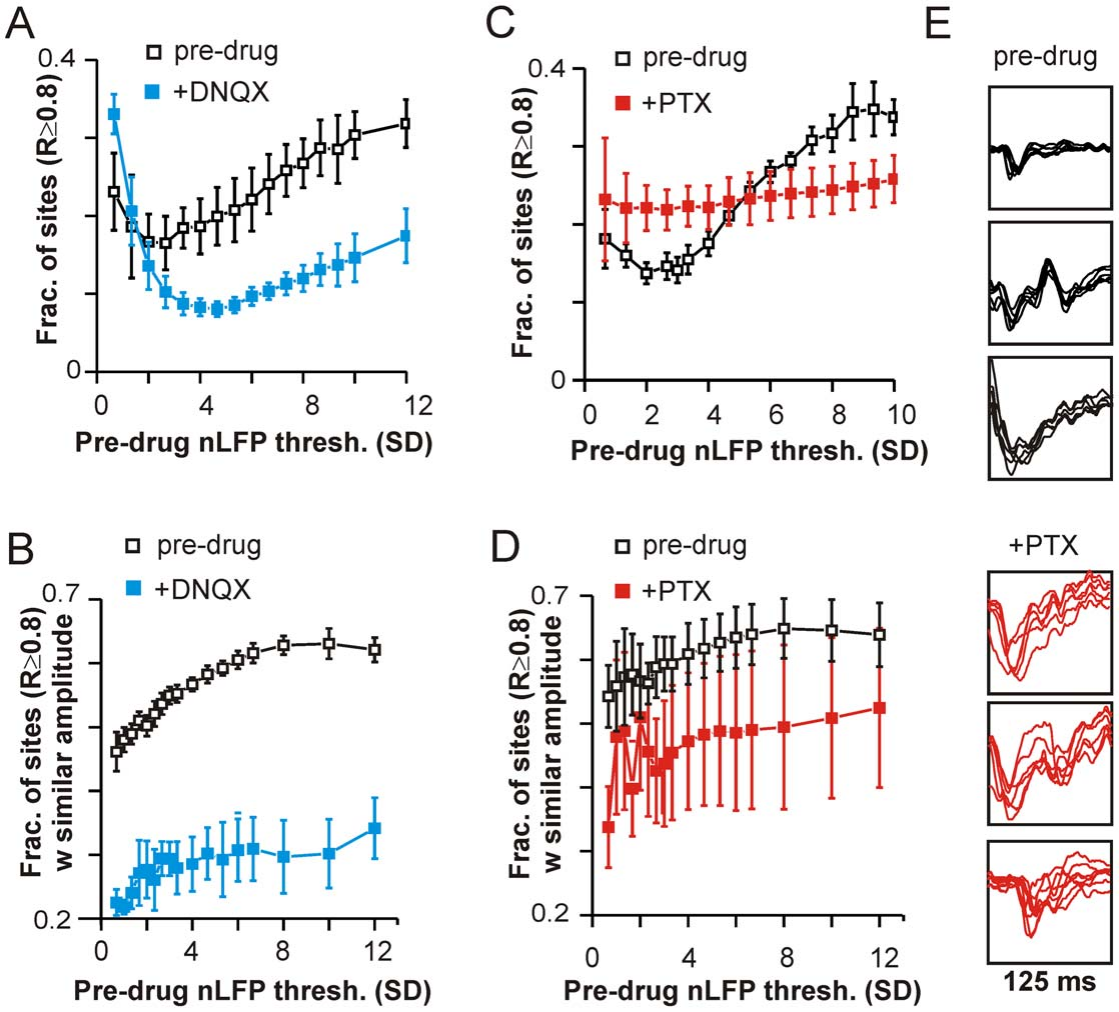
Spatial spread of similar waveforms depends on fast inhibitory and excitatory transmission. (A) Reduced AMPA glutamate-receptor mediated excitation (2 µM DNQX) decreased the fraction of correlated sites at all thresholds (*p*<10^−3^, amplitude thresh ≤−2.5 SD; shown here and in B are the best-match values within a period of ±10 ms). Thresholds used in both cases correspond to the absolute amplitude values before drug. (B) DNQX also significantly reduced the amplitude similarity (i.e., amplitudes within 50% of the selected trigger) of highly correlated waveforms (*R*≥0.8) at all thresholds (*p*<10^−5^). (C) Reducing GABA_A_-receptor mediated inhibition (5 µM Picrotoxin, PTX) destroyed the sigmoidal increase in correlations with increasing amplitude, reducing the extent of correlations at high thresholds and increasing the extent of correlations at low thresholds. (D) Similar comparison as in (B) for *n* = 3 cultures before and in the presence of 5 µM PTX shows that amplitude similarity is also reduced under conditions of reduced fast inhibition (*p*<0.05). In addition, amplitude variability is increased as indicated by the larger error bars. (E) Examples of waveforms with amplitudes ≤−9 SD that are temporally clustered (<10 ms intervals) before (pre-drug) and in the presence of PTX (+PTX) demonstrate the substantial loss in waveform similarity when inhibition is reduced.

The non-linear transition to high spatiotemporal coherence crucially depended on fast excitatory and inhibitory synaptic transmission. In culture, where controlled pharmacological manipulation was possible, reduction of fast excitatory synaptic transmission with 2 µM of the AMPA receptor antagonist DNQX (*n* = 3) reduced the fraction of sites correlated with *R*≥0.8 by over 50%, particularly at higher amplitudes (Figure 6A; *p*<10^−5^ for nLFP triggers ≤−9 SD; note that in order to avoid any distortion due to changes in the LFP amplitude distribution in the presence of the drug, amplitude values used for both pre-drug and drug comparisons are the absolute amplitude values corresponding to pre-drug SDs). Similarly, the fraction of correlated sites with similar amplitude was also dramatically decreased by DNQX application (*p*<10^−4^; Figure 6B). Such a dependency on AMPA-receptor mediated activity indicates that the appearance of waveforms of similar time course and amplitude at different sites is primarily a consequence of fast synaptic transmission and cannot be simply explained by volume conduction, overlapping electrode fields, or electrode filtering characteristics.

Partial reduction of fast inhibitory synaptic transmission also had strong effects, distinct from those of DNQX (Figure 6C–E). Application of 5 µM of the GABA_A_ receptor antagonist Picrotoxin (PTX, *n* = 3, raw trace examples in Figure S9) destroyed the sigmoidal amplitude dependence such that the fraction of sites correlated was no longer dependent on the nLFP trigger amplitude (Figure 6C). The maximal spatial coherence was reduced by ∼30% for high amplitude nLFP triggers (Figure 6C; *p*<10^−2^ for nLFP triggers ≤−9 SD) and concomitantly increased at small amplitudes. In addition, PTX significantly reduced the amplitude similarity of highly correlated waveforms (*R*≥0.8; Figure 6D, *p*<0.05) introducing much greater amplitude variability. Indeed, temporally clustered sequences of high amplitude nLFPs had far lower waveform similarity relative to pre-drug conditions (Figure 6E, cf., Figure 4B). Thus, fast synaptic inhibition is required for the existence of distinct low-coherence and high-coherence regimes.

The finding that waveforms of similarly low amplitude and comparable durations have higher spatial coherence in the presence of PTX indicates that noise, which would be expected to obscure correlations for low amplitude signals, is insufficient to explain the low spatial coherence seen for small amplitudes under normal conditions. Thus, the sigmoidal transition to higher coherence at higher amplitudes cannot be explained by an increase in signal to noise ratio. This is also supported by the large deviation of the overall LFP amplitude distribution from a Gaussian fit both in vitro and in vivo (Figure S10), which suggests that commonly occurring Gaussian noise would have little influence in the range of −2 to −3 SD, where the non-linear transition occurs.

### Coherence Potentials Arising in Rapid Succession Show Sequential Dependence

**Figure 7:**
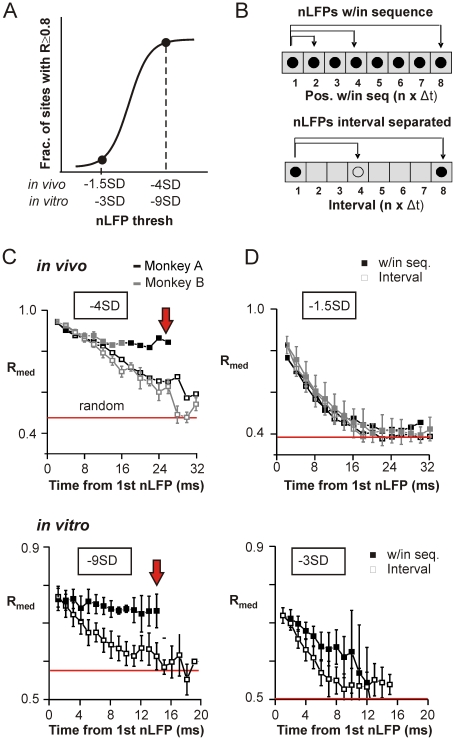
Large amplitude nLFPs propagate stably showing sequential dependence, while small amplitude nLFPs propagate with progressive distortion. (A) nLFP sequences were constructed at two thresholds, before (*left*) and after the transition to high spatial coherence (*right*; cf., Figure 1F). (B) Comparisons were made between the first nLFP and nLFPs whose peaks occurred in consecutive time bins (i.e., in sequence; *top*) as well as between nLFPs separated by time-matched intervals (i.e., empty bins; *bottom*). (C) The median waveform correlation (R_med_) within a sequence of large amplitude nLFPs (≤−4 SD in vivo, *top*; ≤−9 SD in vitro, *bottom*) remained stable relative to the first nLFP (*filled squares*; *red arrow*), while correlations of nLFPs separated by similarly increasing intervals (*open squares*) decayed progressively to the median random value (*random*, *red line*). (M1_left_, monkey A; average over four arrays in monkey B; average over *n* = 6 cultures). (D) R_med_ for sequences of small amplitude nLFPs (*filled squares*; ≤−1.5 SD in vivo, *top* and −3 SD in vitro, *bottom*) decayed relative to the first nLFP to no better than random (*red line*) similar to nLFPs separated by increasing intervals (*open squares*).

The previous section demonstrated that the regime of high spatial coherence reflects a loss-less, undistorted propagation of activity that depends crucially on fast AMPA and GABA_A_ mediated synaptic transmission. We next systematically examined the relationship between successively occurring nLFPs (Figure 7) to determine if there was a sequential dependence that was unique to the high threshold regime of coherence potentials. Evidence for sequential dependence would also give credence to an intrinsic propagation process in vivo, where sub-cortical input was present.

Given that correlated waveforms tended to be nLFPs of similarly high amplitude (Figure 3) and that time delays between sites corresponded closely to peak alignment of the nLFP waveforms (Figure 2B), sequences were defined as nLFPs identified at any site on the array whose peaks crossed a certain threshold and occurred in successive time bins of 2 ms (Figure 7B). For simplicity, sequences with only one nLFP peak in the first time bin were used (>50% of all sequences), although successive time bins often contained multiple nLFPs (cf., Figure S4).

We first examined sequences of nLFP peaks that exceeded a threshold identified by the top of the sigmoid transition to high spatial coherence (Figure 7A; −4 SD in vivo; −9 SD in vitro; cf., Figure 1F). We reasoned that if nLFP waveform similarity across sites arose as a sequential, loss-less process intrinsic to the cortical network, then nLFPs occurring *n* ms apart that formed part of a sequence (Figure 7B, *top*) would be more stable in their waveform similarity across each step in the sequence relative to amplitude-matched nLFPs separated by similar time intervals that were not part of a sequence (Figure 7B, *bottom*). Remarkably, nLFPs within a sequence were highly stable in their correlation relative to the initial nLFP; the median correlation of nLFPs at each position in the sequence relative to the first nLFP decreased only slightly from ∼0.94 to 0.86 in nine time steps in vivo (≈18 ms, Figure 7C, *top*) and from ∼0.78 to ∼0.75 in twelve time steps in vitro (≈12 ms, Figure 7C, *bottom*). In contrast, correlations between nLFPs separated by correspondingly larger intervals were significantly worse than their within sequence counterparts (*p*<0.02 up to *p*<10^−5^ by KS test), decaying to no better than comparisons of random nLFPs of similar amplitude within 15 to 30 ms (Figure 7C, *open squares*; median random correlations were 0.47 and 0.49±0.013 in vivo and 0.58±0.03 in six cultures, *p*<10^−4^ when compared to *within* sequence correlation for all cases). In sharp contrast to this behavior of coherence potentials, sequences constructed predominantly of small or subthreshold nLFPs, i.e., before the transition to the regime of high spatial coherence (Figure 7A), decayed progressively to random in a manner that was no different from interval-matched comparisons both in vivo as well as in vitro (Figure 7D). Thus, the sigmoidal increase in spatiotemporal coherence reflects a transition from a regime of progressive distortion of the waveform time course within sequences to spatially extended sequences that stably maintain waveform amplitude and time course.

In vitro, where there was no source of sub-cortical input, this indicates a clear sequential process intrinsic to the cortical network. In vivo, such sequential dependence is not likely to arise from sub-cortical input and similarly points to cortical propagation. However, we also considered one additional possibility: that subcortical input arrives at different sites on successive cycles of a subcortical oscillation (e.g., thalamocortical spindles) creating an indirect sequential dependence. Inconsistent with this scenario, the time delays between successive nLFPs in *individual* sequences that had at least ten correlated nLFPs (*R*≥0.8) were widely distributed (Figure S11; cf., also Figure 2B) indicating the absence of any characteristic time scale. Thus, all evidence points to a sequential process that is intrinsic to the cortical network both in vivo and in vitro.

### Coherence Potentials Propagate in Saltatory Fashion

**Figure 8:**
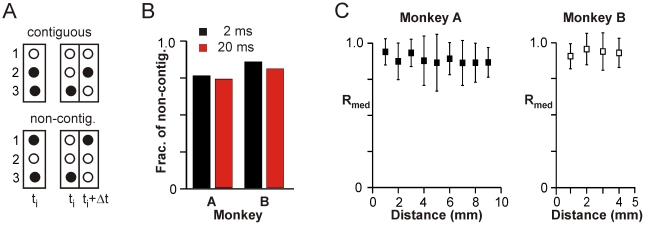
Coherence potentials propagate in saltatory fashion. (A) Sketch of linear 3-electrode array showing an example of spatially contiguous (*top*) and non-contiguous (*bottom*) electrode activations for simultaneous (*left*) or successive (*right*) nLFPs at temporal resolution Δt. (B) More than 70% of coherence potentials in vivo whose peaks occurred either in the same or consecutive time bins were not at contiguous electrodes irrespective of the temporal bin size chosen (Δt = 2 ms and Δt = 20 ms shown). (C) The median correlation R_med_ between pairs of nLFPs in vivo exceeding a threshold of −4 SD was not dependent on the distance between the electrodes on which they occurred. (M1_left_, monkey A; average over four arrays in monkey B).

Propagation in the cortex has been widely described as wave-like. We therefore looked to see if the propagation moved progressively in a wave front. Because time delays between successive coherence potentials were broadly distributed (cf., Figure 2B), it is not possible to determine the true order of occurrence of individual coherence potentials within a large sequence. Thus, we restricted our analysis to cases of coherence potentials where there were only two highly correlated nLFPs either in the same or contiguous time bins. Remarkably, in both monkeys, over 70% of pairs of correlated nLFP waveforms occurring in the same or contiguous time bins were not at adjacent electrodes, irrespective of the temporal bin width (Figure 8A, 8B, shown are time bin Δt = 2 and 20 ms). Moreover, the median correlations of large amplitude nLFPs (≤−3 SD) occurring in the same or adjacent time bins were not different irrespective of the distance separating them (Figure 8C). Thus, there was no evidence of distance dependent distortion. This points, surprisingly, to non-contiguous or saltatory propagation quite unlike a wave, and is clearly contrary to expectations of volume conduction and overlapping electrode fields where similar waveforms would be found at adjacent sites.

### Correlated nLFP Waveforms Have More Correlated Unit Firing Patterns

We have previously shown in the present in vivo data that higher nLFP amplitudes are systematically associated with higher local firing rates as well as a higher number of simultaneously firing neurons or synchrony [35]. This suggests that the amplitude reflects a certain degree of spike synchrony or aggregate spike activity, lending credence to the possibility that particular waveform patterns reflect a particular pattern of spike activity. We thus looked for evidence of a preservation of the underlying pattern of spike activity associated with coherence potential propagation.

**Figure 9:**
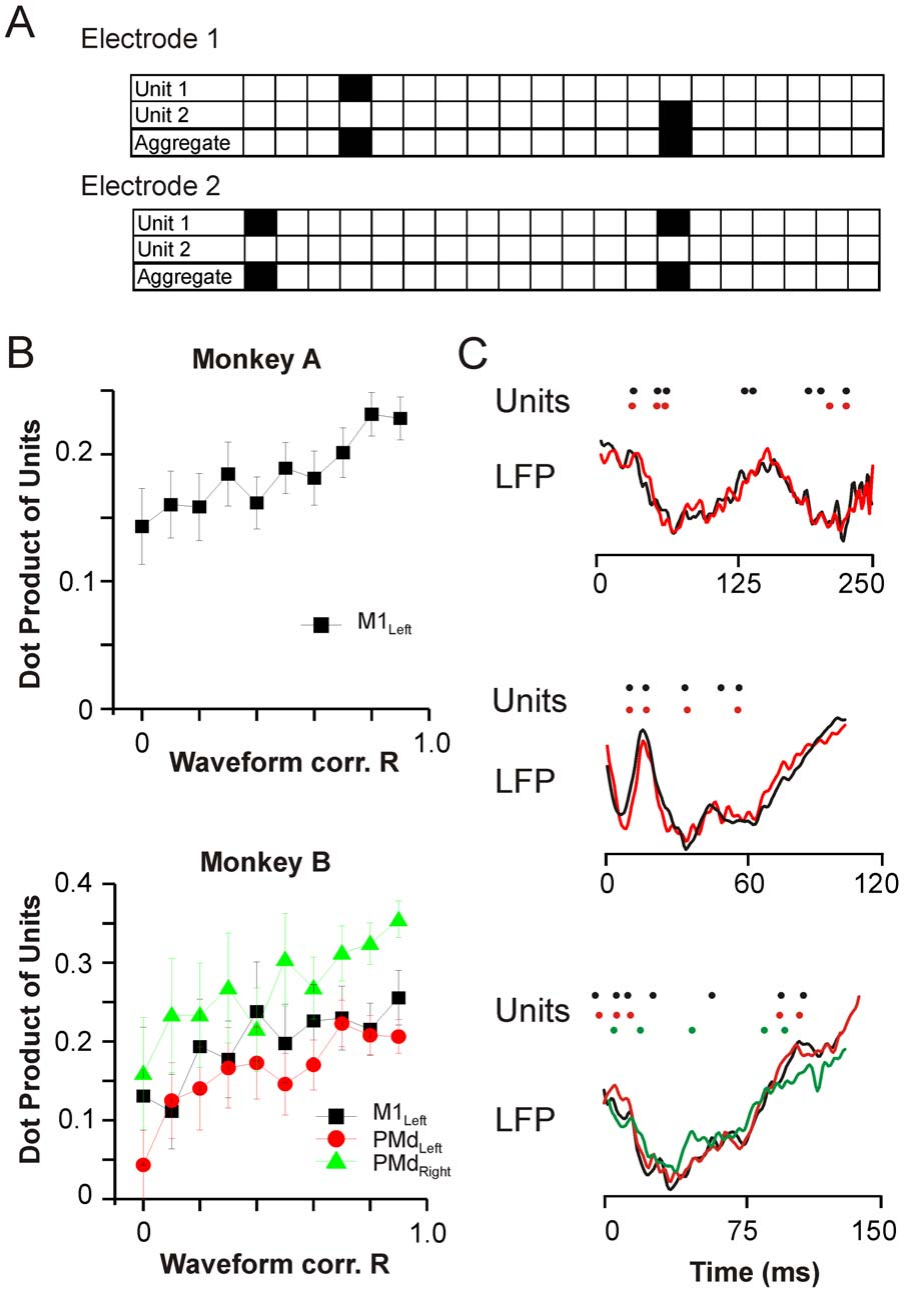
Similarity in temporal pattern of unit activity increases with waveform similarity. (A) Similarity of unit activity was calculated as the dot product of aggregate unit activity around the nLFP peak for different temporal bin widths. To control for duration and number of spikes, periods selected corresponded to coherence potentials of at least 200 ms duration with exactly two unit occurrences. Examples show aggregate unit activity at two electrodes leading to a dot product of 1 for a bin width of 10 ms. (B) Similarity in unit activity correlated positively with LFP waveform similarity in both monkeys. Dot product of aggregate unit activity of pairs of electrodes plotted as a function of the correlation of the LFP waveforms corresponding to the same period and electrodes. (C) Examples of highly correlated nLFPs on two or more electrodes with >3 units each that also had highly correlated unit activity.

While our LFP recordings represent the aggregate activity of several hundred neurons in the local field of the electrode, only a very small number of units close to the electrode can be resolved, representing a very small sampling of the neurons contributing to the aggregate activity. Most electrodes resolved either one or two units, while a small fraction resolved three or four units. Indeed, often there were no units detected in association with a coherence potential indicating a lack of participation of the resolved units. Our analysis was thus necessarily limited to those coherence potentials that had associated unit activity. These were spread across many sequences offering a large diversity of waveforms and therefore waveform correlations within and across different, diverse coherence potentials. We thus made comparisons of the aggregate pattern of unit activity as a function of the similarity of the associated coherence potential waveforms (i.e., nLFPs with peak amplitude ≤−4 SD; cf., Figure 1F). Comparisons were made by calculating the dot product of the summed unit rasters binned at 6 ms controlling carefully for the number of units and duration of comparison, both factors that affect the a priori expectation of similarity (see Material and Methods for details). Indeed we found that the more correlated the coherence potential waveforms at different sites, the more similar the pattern of aggregate unit activity (Figure 9A; linear fit, M1_left_ monkey A: *R* = 0.96, *p*<10^−4^; monkey B: *R*>0.94, *p*<0.005, all arrays). A similar pattern was observed for binning of units anywhere between 2 and 10 ms bins (unpublished data), 6 ms, shown here, represented a midpoint. Some examples of cases with preserved temporal pattern of spikes are shown in Figure 9B.

These findings argue strongly in support of a most unexpected conclusion, that when the activity of many neurons in a local field are sufficiently synchronized, the aggregate activity of these neurons is able to propagate to distant sites without distortion of the overall temporal pattern or substantial change in the number of participating neurons.

## Discussion

Here, we have identified a quasi-discrete network object in the LFP, which we term the *coherence potential* that arises at high amplitudes with a non-linear relationship. A coherence potential is characterized by a complex waveform comprised of negative–positive excursions that exceeds a certain local amplitude threshold and is associated with a synaptic transmission dependent saltatory, cascade-like spread through the cortical network without distortion of its temporal structure or substantial change in amplitude. The transition from a regime where waveforms propagate with progressive distortion (subthreshold potentials) to one without such distortion (coherence potentials) occurs according to a sigmoidal function of LFP amplitude, suggesting a threshold dependent process. This phenomenon could not be explained by enhanced signal to noise at higher amplitudes (Figures 1F, 3, 6, S10), volume conduction, overlapping electrode fields, electrode characteristics (Figures 3, 6, 8), or common input from sub-cortical sites (Figures 6, 7, S11). Rather, its occurrence in both explant cortical tissue and the cortex of awake monkeys suggest that it is an intrinsic property of cortical networks. This has phenomenological parallels with the action potential, which describes a large amplitude potential across a local patch of excitable membrane that represents a non-linear regime in which activity propagates in a loss-less manner. However, unlike action potentials, which are stereotypical waveforms, coherence potentials are highly diverse in their waveforms. Distinct coherence potentials, formed of sequentially occurring nLFPs, often occurred in rapid succession as a stream of dynamical associations (Figure 4) representing an identifiable switching of the cortical network from one dynamical state to another.

### Coherence Potentials, Waves, Synfire Chains, and Neuronal Avalanches

The propagation of coherence potentials was saltatory in nature, rather than wave-like, frequently “jumping” across neighboring sites (Figure 8B) such that the fidelity of propagation was not substantially impacted by physical distance (Figure 8C). How might this phenomenon be reconciled with wave-like propagation ubiquitously reported in the cortex using techniques ranging from voltage sensitive dye imaging to LFPs and spikes recordings [10],[42],[43]? While this remains to be clearly understood, we provide a hypothetical framework that could reconcile these two findings. Coherence potentials arise at high thresholds of −4 SD in vivo and −9 SD *in vitro*, thereby representing only a very small fraction of nLFP activity in the cortex (<1%). This means that most of the activity one measures is sub-threshold in nature. Thus, in the absence of knowledge of a threshold dependent process of loss-less propagation, one would include both sub-threshold and supra-threshold activity, whereby alternative dynamics, such as wave-like propagation, might dominate. We therefore suggest that sub-threshold network activity may spread in a wave-like manner analogous to the spread of a sub-threshold depolarization in a neuron. We note that both waves and coherence potential propagation depend crucially on inhibition [41]. It will be interesting in the future to understand the mechanistic similarities and differences.

The amplitude dependence of the transition to loss-less propagation indicates that coherence potentials are characterized by a certain level of synchronization in the local field [35]. Theory has suggested that synchrony might propagate differently from single spikes. For example, synfire chain models in feed-forward networks report that the size of the initial synchronized group determines propagation fate in a threshold dependent manner [44],[45]. In these basic models, however, there is convergence to a simple waveform as activity propagates, which is in contrast to coherence potentials, which can be highly diverse in their waveforms, and where the original temporal properties of the waveform or underlying synchronized group are preserved in the propagation to other sites. This interpretation equates the time course of a local nLFP with the synchronization of inputs arriving at a single layer of a synfire chain and where the coherence potential itself, which spatially extends over different sites, would reflect the various, spatially distinct layers of the chain. Alternatively, and in line with the general argument that only local connectivity between pyramidal neurons is high enough to sustain a synfire chain [44],[46], the nLFP time course could reflect the synchronization time course of a local synfire chain. In that case, coherence potentials would reflect phase-locking of distant synfire chains as suggested in studies on compositionality by simulating several chains that are weakly coupled through long-range connections [47].

Additionally, once recruited in this manner, sites may become locked into recurrent interactions that “attract” each site to a common pattern of activity for a short period of time. Such an interpretation is consistent with the long durations of the coherence potentials (several tens to hundreds of milliseconds) relative to the rapid time scales of propagation (generally a few milliseconds) and is supported by the high degree of reciprocal connectivity observed in cortical networks [48]–[51]. This interpretation has parallels to the principles underlying the construction of attractor neural networks or Hebbian cell assemblies [1],[52]. Evidence for recurrent interactions has also been described in the propagation of waves across cortical areas [53]. Such inter-areal phenomena also raise interesting questions about how propagation of coherence potentials across cortical regions may occur and how coherence potential originating at different cortical regions may interact.

Finally, we note that in each of the datasets used in this study, nLFPs have been shown to organize as neuronal avalanches [35],[54]. Neuronal avalanche organization is a statistical property characterized by scale invariance in the temporal and spatial clustering of nLFPs suggesting that propagation could in theory span the entire cortex on a wide range of time scales [55]. Furthermore, partial reduction of fast GABAergic transmission abolishes the neuronal avalanche dynamics [35],[54] as well as the sigmoidal dependency of coherence potentials (cf., Figure 6C). It is thus of considerable importance to further understand the sigmoidal, non-linear transition to the coherence potential regime that reflects propagation without distortion and the general scale-invariant organization of nLFPs in the context of neuronal avalanches that may govern the organization of successive coherence potentials.

### Mechanism Underlying Coherence Potential Propagation

The greater similarity of aggregate unit firing patterns at sites with similar LFP waveforms (Figure 9) suggests that coherence potential propagation reflects a preservation of the temporal characteristics of the underlying neuronal activity. Given that electrode noise and the spatial arrangement of the participating neurons relative to each electrode contribute to differences in waveforms observed at distinct sites [56], the precision with which coherence potentials propagate in the network might be even higher than we measured. The amplitude dependence and the initiation of high correlation periods on the rise phase only milliseconds before the peak (Figure S7) suggests that it is the synchronization of activity itself that acts as the trigger. Indeed, at the level of the individual neuron, increasing synchronization of input gives rise to a non-linear increase in precision and reliability of the input-output relationship by altering the threshold for spike formation [57]–[60]. Computational models also suggest a crucial role for fast inhibition in regulating the precision and reliability of output [61]–[63], consistent with the dependence of coherence potential propagation on GABA_A_ dependent transmission. However, even with these findings at the single cell level, it remains to be understood how locally, synchronized activity can be directed to another site in an all-or-none manner.

### Coherence Potentials and Oscillations

Unlike the stereotypical action potential, which can serve only as a binary code, the diversity of coherence potential waveforms represents a high dimensional degree of freedom, which could be used to encode information. In contrast, coherence potentials demonstrate the maintenance of spectrally complex waveforms and therefore indicate phase-locking or spectral coherence between sites across a broad range of component frequencies. While coherence potentials in our data showed no dominant frequency, this does not preclude them from exhibiting clear oscillatory components in the waveform under certain stimulus or behavioral conditions. Indeed in a small fraction of cases, we did find an oscillatory component (Figure S8). We propose that oscillatory behavior may represent a certain class of coherence potentials, analogous to a burst of action potentials, where the duration of waveform correlation may be extended across a greater number of positive–negative cycles. This proposition, of course, must be tested empirically but points to a new approach to LFP analysis, where, by not restricting analysis to a particular frequency band, it might be possible to make finer distinctions in the information contained in the complex structure of the LFP waveforms.

### Possible Functional Implications of Coherence Potentials

A fundamental question that arises is how to reconcile the occurrence of similar associations both in stimulus deprived explant networks and the awake animal in a functional context. Here we suggest that just as the action potential arises by virtue of the intrinsic properties of the cell in a dish but takes on meaning in the intact organism by virtue of an input-output feedback mechanism, coherence potentials, which likely represent an aggregation of spiking activity, arise by virtue of intrinsic properties of the cortical network and take on meaning in a similar fashion. That temporal properties of the LFP waveform often serve as better predictors of behaviors than spikes [10],[12]–[15],[64]–[66] is consistent with such an explanation, suggesting that coherence potential propagation in vivo represents the transfer of meaningful information in the cortical network.

Sequences of propagated coherence potentials occurred in rapid succession as a stream of dynamical associations (Figure 4) representing an identifiable switching of the cortical network from one dynamical state to another, which is a hallmark of “metastability” [8],[27]–[34]. However, the question remains how the cortex might make use of the specific phenomenon of coherence potentials, where many sites mirror one another with millisecond delays, reflecting transmission of information without any distortion. One possibility is that such propagation may serve as a form of working memory. However, to spark debate and discussion, we put forth a more radical hypothesis, drawing from the analogy to the spread of ideas, innovation, and economic behavior in social networks, which tend to display a threshold or “tipping point” such that a small increase in the number of participating agents can suddenly lead to widespread cascades of adoption [67],[68]. The initial adoption of the idea or behavior often arises among geographically proximal agents [69],[70] on networks with small-world principles [71] similar to those observed in cortical networks [72]–[75]. “Tipping points” are commonly modeled under conditions where individual agents make binary decisions (e.g., to buy or not to buy) by integrating the incoming information from their local neighborhood of friends [76], in close parallel to the “integrate and fire” property of neurons. We propose that perhaps information competes to pervade the cortex analogous to the way that ideas compete to pervade society, originating from various sensory areas in the way that ideas arise in society from specialized regions or areas focused on particular subjects or activities. Perhaps it is the case that when a sufficient number of sites across multiple cortical regions have adopted a particular module of cortical information, this now has the ability to impact the behavior of the organism. Such a hypothesis is not entirely unwarranted. For instance, coordinated cortical activity has been shown to be associated with motor behavior [10],[17], the spread of voltage-sensitive dye signals from sensory to motor areas of cortex and their amplitudes have been found to be correlated with animal whisking behavior [77], and the diversity found in average LFP waveforms across many electrodes have been shown to encode behaviors [78]. We note that the diversity of coherence potential waveforms in vivo was much greater than in vitro indicating a richer associative environment and we suggest that a comprehensive characterization of coherence potential waveforms and their spatial spread in relation to behavior is likely to be highly instructive with respect to a broad range of cognitive functions.

## Materials and Methods

### In Vivo Recordings in Non-Human Primates

The ACUC of Duke University approved all procedures. Arrays of monopolar tungsten electrodes (30 µm in diameter, 1 MΩ impedance; 1 mm spacing) were chronically implanted into the cortex of two adult rhesus monkeys (*Macaca mulatta*) [79]. Briefly, arrays were inserted 1.5 mm deep into the leg representation area of the motor cortex (M1_left_) and 1 mm deep in the somatosensory cortex (S1_left_) in the left hemisphere of monkey A. In monkey B, four arrays were similarly implanted in the arm representation of the left and right motor cortex (M1_left_, M1_right_) and dorsal premotor cortex (PMd_left_, PMd_right_) (for sketch in electrode arrangements and array positions, see [35]). During the ∼40-min-long recording sessions analyzed here, the monkeys were awake and seated in a monkey chair with the light in the recording room turned off. LFPs were sampled at 500 Hz (National Instruments) and band-pass filtered between 1 and 100 Hz using the idealfilter function in MATLAB. Results were subsequently verified for M1_left_ in monkey A using a phase-neutral filter (MATLAB filter function “filtfilt,” Figure S12). No difference was found between the two filter approaches.

Extracellular spiking activity was sampled at 40 kHz and band-pass filtered with a 2-pole low-cut and a 4-pole high cut filter at 0.4–8 kHz. Off-line unit discrimination was based on principal component analysis and spike-template matching (for details, see [79]). Simultaneous recordings were carried out in M1_left_ and S1_left_ (monkey A) and the four arrays in M1 and PMd (monkey B). LFP and unit activity was sampled simultaneously from every other electrode resulting in 32 electrodes for M1_left_ and S1_left_ for monkey A and 16 electrodes per array in monkey B using a dual amplifier Plexon system (Figures 1A and S5; filled circles). An epidural stainless steel T-bolt, at least 20 mm from all recording areas, served as a common ground (for a detailed sketch of electrode location and array positions, see [35]). The 68 units resolved in M1_left_ (32 electrodes) fired on average at 4.3±3.7 Hz. For the four arrays in monkey B, on average 47±13 units were resolved (16 electrodes/array), which fired at a rate of 5.7±1.8 Hz. Unit firing rates were not significantly different between monkey A and monkey B (Student's *t* test, *p* = 0.3).

Some periods in the recording exhibited higher amplitude, slow-wave activity that was characteristic of sleep-spindles (90% of power at 1–10 Hz [80]); periods with >50% channels showing this type of activity accounted for 6% to 12% of the recordings in monkeys A and B, respectively, and contributed between 5.5% and 9.5% of high amplitude nLFPs (≤−3 SD), not substantially different from the rest of the recording.

### In Vitro Organotypic Slice Cultures

Organotypic cortex slice cultures were prepared as previously described [36],[54]. Briefly, coronal section of somatosensory and prefrontal cortex from postnatal day 1–2 old rats were grown on planar integrated 60-channel microelectrode arrays (8×8 grid; 30 µm diameter titanium nitride electrodes; Multichannel Systems). One enlarged electrode served as the common ground. At an electrode spacing of 200 µm as used in the present study, there is no detectable overlap between electrode fields [81]. After 4–6 wk of cultivation, spontaneous LFP activity emerged and was recorded for up to 5 h and filtered between 1 Hz and 50 Hz. To study the effects of changes in synaptic transmission, after 2 to 5 h of baseline recordings, either DNQX or picrotoxin (PTX) was added directly into the medium of the cultures (final concentration 2 µM and 5 µM, respectively) and recordings were continued for 2 to 5 h.

### Correlation Analysis and Controls

Correlation analysis was carried out using in turn each electrode as the trigger electrode and selecting a large number of nLFPs (*n*≥250) of each amplitude from the recording on this electrode as triggers. Correlations *R* were then calculated between the nLFP trigger (X) and a period of equal duration (Y) on each other electrode as
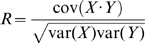
where 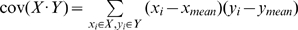 and *var* indicates the variance of the signal. To prevent double counting of nLFPs, the distributions of correlations were obtained for each trigger electrode and the fraction of sites correlated *R*≥0.8 were averaged across electrodes for Figure 1F. The controls (Figure 1B, E, F) were done as follows. To control for random similarity (Figure 1B, E, F; *red*), a random period of similar duration to the nLFP trigger was selected by beginning from a random position chosen from anywhere in the recording that was at least 100 ms away from the peak of the nLFP trigger. To control for the large variability in the duration of trigger nLFPs (Figure 1D), a factor that could greatly influence correlations, we generated random time-aligned and time-shifted comparisons across electrodes that had the same distribution of durations as the nLFP triggers but were *not* nLFP triggered. Because these were not nLFP triggered, they controlled only for the durations seen at particular nLFP amplitude thresholds (Figure 1C, E, F; *green*) and not the amplitudes themselves. To control for the possibility of an overall greater similarity between large amplitude deflections (Figure 1F; *blue*), for each nLFP trigger we made a comparison to an amplitude-matched nLFP at each other electrode and then identified the fraction of cases that were ≥0.8. To do so, all nLFPs arising on the comparing electrode that were of comparable amplitude (within ±10%) and whose peaks were at least 100 ms away from the nLFP trigger peak were identified and a random selection was made from this group. Because amplitude-matched nLFPs were often of different durations, the period of comparison was defined as the duration of the trigger nLFP before and after its peak. For the controls in Figure 7, all nLFPs of comparable amplitude on all electrodes were pooled together and 500 randomly selected pairs were compared, creating a distribution of random correlations for different interval times. In this case, variability of nLFP duration was dealt with by using segments corresponding to the length of time from the first start to the last end of the nLFP pair.

### Time-Shift Analysis

Time-shift analysis (Figures 1B, 1F, 2A) was carried out by moving the window of comparison on each comparison electrode one time bin at a time in both directions relative to the nLFP trigger window position (bin width Δt = 1 ms in vitro, 2 ms in vivo) and identifying the shift corresponding to the highest correlation. For the random and duration controls, the window was similarly shifted relative to the originally chosen random position. Intervals between successive correlated periods were calculated by assuming a one-to-one sequential process (Figure 2C). For example, if there was one nLFP in the first time bin and three correlated nLFPs in the second time bin, the inter-event intervals would be counted as 1×Δt between the first nLFP and the first nLFP in the second time bin (nLFP order was arbitrarily chosen based on electrode number) and 0 between the second and third nLFP and third and fourth nLFP in that second time bin, giving a distribution of delays of 2 zeros and 1 Δt.

### Amplitude Similarity

In order to quantify amplitude similarity as a function of nLFP trigger amplitude (Figure 3), peak amplitudes were identified as the minimum within the time-aligned window of comparison. Since the duration of the nLFP, which set the window of comparison, was generally many tens to hundreds of milliseconds, and since best-match time delays were usually <50 ms (Figure 2), this ensured that in most cases, the true peak was identified. The peak amplitude of the comparison period was then normalized by the peak amplitude of the nLFP trigger.

### Visualization of Coherence Potentials

Best-match correlations and peak-to-peak time differences were calculated for each pairwise combination of 100 (Figure 4A, B) and 30 (Figure 4D, E) nLFPs ≤−3 SD whose peaks occurred on the array during a 15 s and 250 ms period, respectively. Because nLFP periods were of variable duration, correlations were calculated for the period corresponding to the maximal nLFP duration preceding and following the nLFP peak of the nLFPs in the pair. Over-plots shown begin 50 ms before the peak crossing to 250 ms after the peak in order to ensure the plots are peak aligned and to encompass the complete duration of the longest nLFP. Note that most of the nLFPs were much shorter than 300 ms, while the matched period in the over-plots often extended beyond the nLFP indicating periods of high correlation beyond the nLFP.

### Power Spectrum Analysis

Power spectrums of coherence potentials with negative deflection periods of at least 256 ms in length were calculated using the Fast Fourier transform (FFT, MATLAB) for a period of exactly 256 ms (Figure 5D). The PSD was also calculated using the FFT of the autocorrelation function with similar results (see Figure S8). Phase dependency of coherence potentials was studied (Figure 5B) by calculating the FFT of the original coherence potentials, shuffling phase angles, and obtaining a new LFP time course using the inverse FFT. Correlations were obtained for original coherence potentials and corresponding phase-shuffled waveform periods.

### Unit Activity Correlation with Coherence Potentials

Several controls and considerations went into this analysis (Figure 9). In order to ensure that we were working with the maximal amount of unit data given the very small sampling of units relative to the population contributing to the LFP, we made comparisons of the aggregate or summed unit activity at each electrode rather than individual units. For each electrode, this was done simply by summing the number of units in each time bin Δt resulting in a single unit activity vector for each electrode. At bin widths Δt = 2–10 ms used here (Δt = 6 ms shown), these vectors mainly contained values of 0 and 1, occasionally the values 2 and 3. The dot product between two vectors was used to quantify the similarity in unit activity patterns between electrodes. Because coherence potentials varied in duration, corresponding unit activity vectors varied in their number of time bins. In addition, the number of units detected between electrodes also varied, affecting the a priori expectation of empty time bins. Since duration and number of units impact the a priori expectation of the dot product, i.e. similarity, it was essential to precisely control for these factors. We kept the duration of comparison constant by choosing only those coherence potentials that were at least 200 ms in duration and comparing the period starting 50 ms before the nLFP peak until 150 ms after the peak. We further restricted the comparisons to only those cases where there were *exactly* two unit occurrences during this period (presumably two spikes, *n* = 520 in monkey A and *n* = 200–400 in monkey B for 3 out of 4 arrays. In the remaining array of monkey B there were *n* = <100 cases, which was inadequate for the analysis across the range of coherence potential correlations). An example with a dot product of one is shown in Figure 9A. Single unit occurrences were excluded from analysis because it did not allow conclusions about temporal structure, while three or more unit occurrences were too rare for statistically relevant aggregation (<75 in all cases).

### Statistical Analysis

For statistical significance between dissimilar distributions, the KS test was used. For curve fitting, regression analysis was performed using a standard linear or sigmoidal function (Origin). Significance was established at *p*<0.05.

## Supporting Information

Figure S1 **Examples of correlations for pairs of nLFP waveforms.** Examples of nLFP pairs with increasing correlation (top to bottom) visualize how similarity increases with increasing coefficient of correlation. Identical y-scale for each waveform comparison.
(0.81 MB TIF)

Figure S2 **Sigmoidal increase in highly correlated sites for ongoing activity in somatosensory cortex of monkey A.** (A) Analysis of correlated nLFPs in somatosensory cortex (S1_Left_) of monkey A. The array consisted of sixteen electrodes inserted about 1 mm into the cortex. The average fraction of highly correlated sites across suprathreshold nLFPs (i.e., *R*≥0.8, shaded area in Figure 1E) increased sigmoidally with nLFP amplitude beyond ∼1 SD (sigmoidal fit, *R*>0.99) and was significantly different from all controls (*red*, *green*; *p*<10^−4^ all cases). For further information on controls, see Figure 1 and main text. (B) The distribution of temporal differences between highly correlated nLFPs (≤−3 SD; *R*≥0.8; best-match comparison) centers around 0 as found for other cortical areas in vivo and in vitro (cf., Figure 2B). The distribution is similar for peak-to-peak time differences (*red*).
(0.38 MB TIF)

Figure S3 **Distribution of time differences was similar between all electrode pairs.** The distributions of time differences between coherence potentials on each pair of electrodes on the array are similar to each other. Shown here are the comparisons between one electrode and every other electrode on the array for the M1_Left_ region of monkey A. The lack of any characteristic differences between any electrodes suggests that the time differences arise due to functional parameters rather than structural or anatomical differences due to electrode positioning.
(0.18 MB TIF)

Figure S4 **Coherence potential propagation is consistent with a cascade model.** (A) A cascade model of propagation where activity originates from a single site and propagates in a one-to-many fashion for successive time bins. (B) Consistent with a one-to-many process, the first time bin had a much lower likelihood of having multiple nLFPs compared to the next two time bins. Graph shows fraction of cases in each successive time bin where there was more than one correlated nLFP. Analysis uses only nLFP sequences extending to ten or more sites (M1_left_, monkey A; average over four arrays in monkey B; average over *n* = 6 cultures). (C) The probability of propagating to *n* sites, approximated by the number of nLFPs in the (n+1)^th^ time bin relative to the n^th^ time bin, decreased according to an exponential function (0.83e^(−n/1.7)M^, *R* ^2^ = 0.98 in vivo and 1.2e^(−n/1.2)^, *R* ^2^ = 0.99 in vitro). Same legend as in *B*.
(0.34 MB TIF)

Figure S5 **Correlated periods are similar in peak amplitude.** (A) Normalized peak amplitude comparisons as in Figure 2F–2H. (B) Fraction of sites with peak amplitude within ±50% of the nLFP trigger amplitude for the somatosensory array (S1_Left_) in monkey A increased non-linearly as a function of trigger amplitude and was significantly greater for correlated waveforms alone. (C) Similar result as in *B* for monkey B (average over four arrays). (D) Similar result as in *B* for cultures (average over *n* = 6 cultures).
(0.53 MB TIF)

Figure S6 **Raw traces of periods encompassing one or more coherence potentials.** (A) Raw traces of three 750 ms periods encompassing one or more coherence potentials from all thirty-two electrodes in M1_Left_ in monkey A. (B) Blow up of the first 750 ms period (*left*). Same 750 ms period after rearranging the electrode order (*right*). Rearranged panel shows five distinct coherence potential sequences (*red boxes*) spanning different subsets of electrodes, providing demonstration of successive within and between sequence comparisons. (C) Raw traces of all 60 electrodes corresponding to one 750 ms period from one culture encompassing one or more coherence potentials. (D) Blow up of signal during period corresponding to box in *C* for select electrodes, with order rearranged.
(1.54 MB TIF)

Figure S7 **Correlated periods are initiated at large amplitude crossings.** (A) The onset of maximal waveform correlation preceding the first threshold crossing (*preceding thresh*) and following the last threshold crossing (*following thresh*) was identified by progressively increasing the window of comparison in each direction. Because coherence potentials almost always included both a positive and negative excursion in a yet undetermined order, we considered threshold crossings in both directions. As our first amplitude threshold (*thresh 1*) we chose the first value that gave rise to the maximal fraction of correlated sites (cf., Figure 1C; ±4 SD in vivo and ±9 SD in vitro). (B) Distributions of durations (identified as in *A*) correlated with *R*≥0.8 preceding the first crossing of threshold in either the positive or negative direction (thresh 1: ±4 SD in vivo, ±12 SD in vitro; thresh 2: ±1 SD in vivo, ±3 SD in vitro). In monkey A, while ∼50% began at or *after* the first crossing of 4 SD, only ∼6% ended at or *before* the last threshold crossing. The pattern was similar for monkey B (22±2 ms preceding, 76±13 ms following) and in vitro (11±4 preceding, 65±41 following) indicating that while coherence potentials were initiated shortly before a large amplitude crossing, they could continue well beyond the last crossing. Of these, ∼60% in vivo and ∼72%±3% in vitro were initiated by negative amplitude crossings while the remaining fraction was initiated by positive amplitude crossings, indicating the presence of an inverse pattern, i.e. negative-positive and positive-negative. This lack of clear directionality likely reflects the positioning of neuronal activity relative to the electrodes and therefore whether the electrode measures the sink or source of the activity but may also reflect differences in the arrival of excitatory and inhibitory input or relationships between spike occurrence and the associated synaptic currents. (C) 75^th^ percentile values of durations from distributions constructed as in B for each of monkeys 1 and 2 and the average of six cultures. The 75^th^ percentiles of the durations (Figure 5C, *left*) preceding the first threshold crossing and following the last threshold crossing were 33 ms and 136 ms, respectively. The short duration preceding the threshold crossing suggests that the correlations begin during the negative or positive rise of the LFP. When the threshold was lowered to 1 SD in vivo and 3 SD in vitro (*thresh* 2, right), the fraction of correlated periods initiated at or after the first crossing of thresh increased to over 80% in all cases (75^th^ percentiles are thus<resolution of the recording), while correlations extended for 20 to 30 ms after the last crossing. Since >90% of durations included only one baseline crossing, this suggests that the periods of correlations are detected as soon as the deflections exceed noise and continue to include most of the succeeding positive or negative deflection.
(0.46 MB TIF)

Figure S8 **Power spectrums and autocorrelations for individual coherence potential waveforms.** (A) Power spectrums (in log-log coordinates, shown from 10–100 Hz) calculated as the FFT of the autocorrelation for forty distinct, randomly selected coherence potentials ≥200 ms in duration. The power spectrum is broad and generally decays without any dominant frequency. (Note that this method is different from that shown in Figure 4 but is in agreement with the findings where the FFT was applied to the signal itself.) (B) Examples of power spectrums (in log-log coordinates, shown for 1–100 Hz) for periods extending ±4 s from the nLFP peak do not show any clear characteristic low frequency. (C) Autocorrelations of the coherence potentials in *A*, obtained by taking the nLFP period and calculating its correlation to time shifted segments of equivalent duration from the same signal. For all cases, autocorrelations were calculated up to 50 ms preceding the peak of the nLFP and 350 ms after the peak using corresponding time segments around the coherence potentials. Autocorrelations were highly variable with a small subset revealing an oscillatory component (marked with a *).
(1.31 MB TIF)

Figure S9 **Raw traces of in vitro recordings before and after application of 5 µm picrotoxin (PTX).** (A) Period of spontaneous neuronal activity in a single cortex culture. (B) Spontaneous activity in the presence of 5 µM of the GABAA-receptor antagonist PTX, which reduces fast synaptic inhibition (same culture).
(1.06 MB TIF)

Fiugre S10 **The LFP signal deviates from Gaussian noise at large amplitudes.** (A) Two second long traces of local field potential (LFP) activity recorded from two electrodes of one in vivo array (*left*) and one in vitro array (*right*). (B) Histograms of the LFP amplitudes at each electrode (*gray*) and average (*black*). Amplitude scale is in multiples of standard deviation (×SD) of the average activity for a representative array in vivo (*left*) and in vitro (*right*). Ordinate axis is shown in log units to emphasize tails which deviate significantly from the best Gaussian fit (*red*). Gaussian fit is closely mirrored by instrument noise measured at the reference electrode (blue, in vitro only). The deviation of amplitudes from the Gaussian fit occurred roughly in the range of 0.75 SD (in vivo) to 1.5 SD (in vitro), indicating that beyond these values the behavior of the signal was likely to be highly distinct from noise. (C) Average LFP amplitude histogram for each of the four arrays in monkey B (*left*) and six arrays in vitro (*right*) show similar patterns.
(0.76 MB TIF)

Figure S11 **Intervals between successive nLFPs of individual coherence potentials. Distributions of intervals between successive highly correlated nLFPs (i.e., coherence potentials).** Shown are forty such coherence potentials spanning >10 electrode sites (individual boxes). Wide range of intervals or delays indicates no characteristic time scale. This result is inconsistent with coherence potentials arising on different cycles of an oscillation.
(0.68 MB TIF)

Figure S12 **Comparison of forward and forward-backward filters for monkey A.** (A) Segment of recording at sixteen of thirty-two electrodes from monkey A (M1_Left_) filtered with a forward filter (“idealfilt” in MATLAB) and a phase neutral forward-backward filter (“filtfilt” in MATLAB) shows high similarity. (B) Sigmoidal transition to a regime of high spatial coherence (time-aligned analysis as in Figure 1F for M1_Left_; monkey A) does not depend on the filter method. (C) Corresponding probability density of time intervals between successive, correlated sites (see Figure 2B main text) does not depend on the filter method.
(0.39 MB TIF)

## Abbreviations

FFT: Fast Fourier transform
KS: Kolmogorov-Smirnov
LFP: local field potential
nLFPs: negative deflections in the LFP
PSD: power spectrum density
