# Correction: Coherence Potentials: Loss-Less, All-or-None Network Events in the Cortex

**DOI:** 10.1371/annotation/f6193dfe-a90c-4110-b0a0-22d81a87b921

**Published:** 2010-01-12

**Authors:** Tara C. Thiagarajan, Mikhail A. Lebedev, Miguel A. Nicolelis, Dietmar Plenz

There was an error in the third sentence of the second paragraph of the section "Correlated nLFPs Are Temporally Clustered with Millisecond Delays". It should read: "Note that the maximal average fraction of correlated sites as a function of trigger amplitude is achieved when there are >50% available sites on the array, and thus the upper part of the sigmoidal function cannot be explained by a ceiling effect due to limited array size."

