# Correction: Coherence Potentials: Loss-Less, All-or-None Network Events in the Cortex

**DOI:** 10.1371/annotation/668be458-400b-4c19-accf-34fc543340e2

**Published:** 2010-01-12

**Authors:** Tara C. Thiagarajan, Mikhail A. Lebedev, Miguel A. Nicolelis, Dietmar Plenz

The first sentence of the section "Coherence Potentials and Oscillations" is in error. Instead of the current sentence it should read: "Oscillations in γ- and β- frequencies in the LFP have long been associated with both behaviors and neuronal synchronization over long distances of the cortex [16-19]."

