# Supplementary figures and images for: Coherence Potentials: Loss-Less, All-or-None Network Events in the Cortex
Source: PLoS Biol. 2010 Jan 12;8(1):e1000278. doi: 10.1371/journal.pbio.1000278 (PMC2795777; doi:10.1371/journal.pbio.1000278)

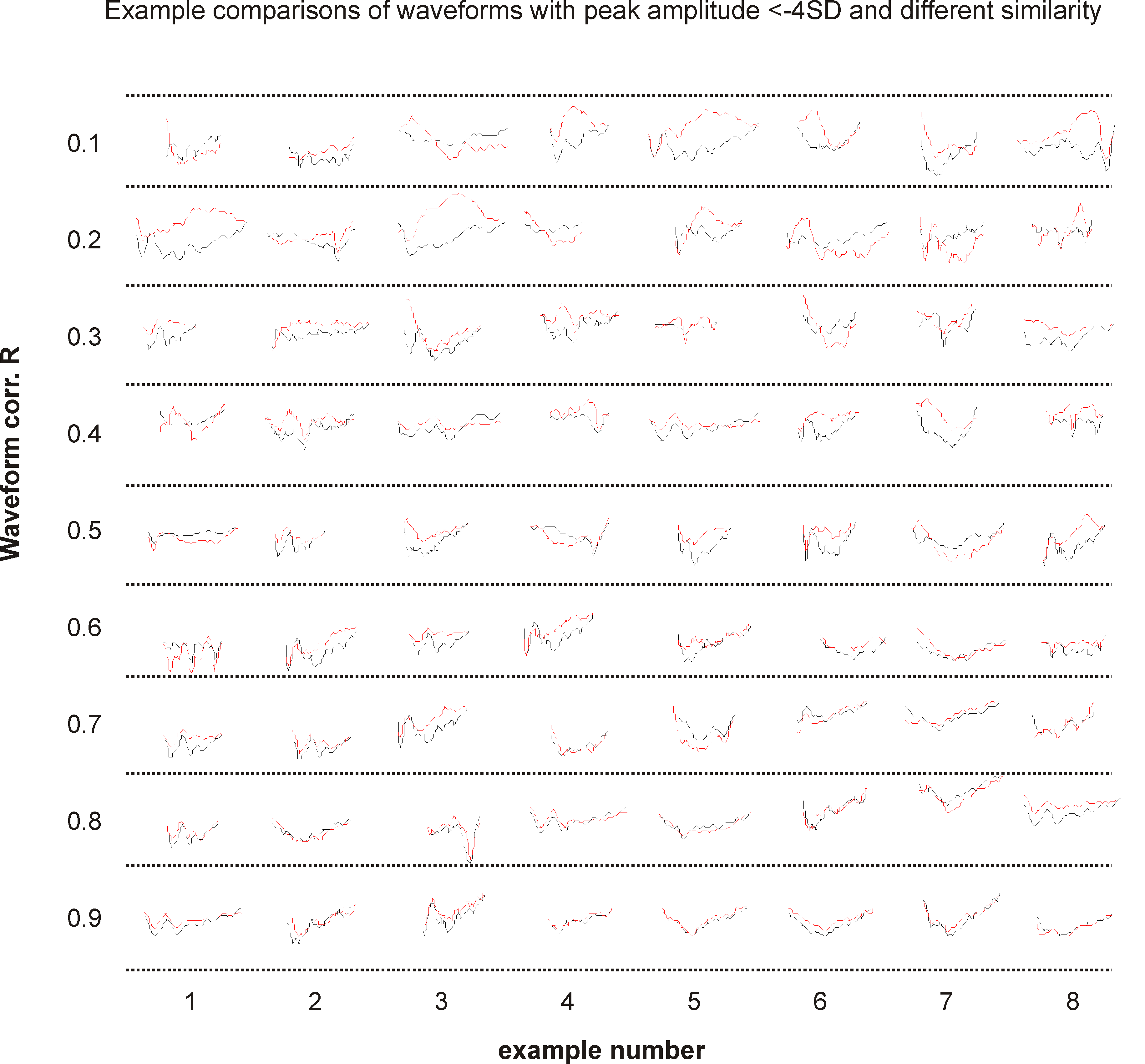

Supplement: Figure S1 — Examples of correlations for pairs of nLFP waveforms. Examples of nLFP pairs with increasing correlation (top to bottom) visualize how similarity increases with increasing coefficient of correlation. Identical y-scale for each waveform comparison. (0.81 MB TIF) [file pbio.1000278.s001.tif]

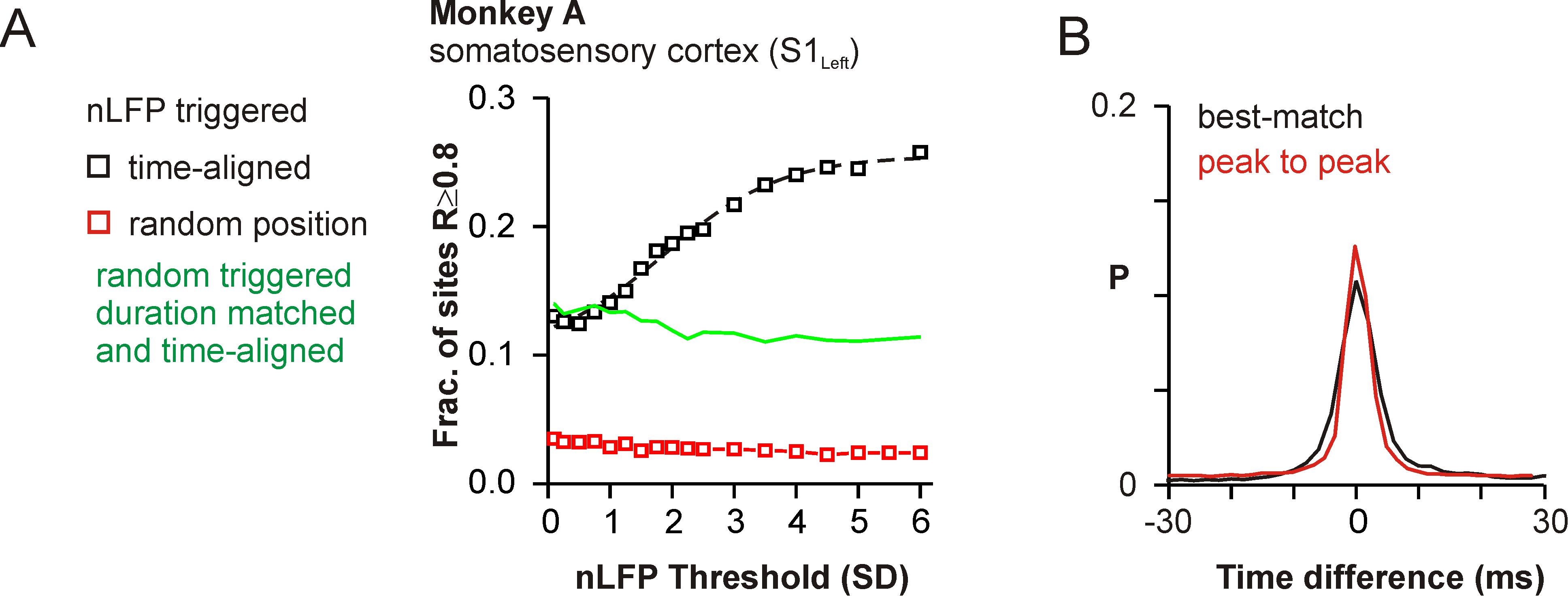

Supplement: Figure S2 — Sigmoidal increase in highly correlated sites for ongoing activity in somatosensory cortex of monkey A. (A) Analysis of correlated nLFPs in somatosensory cortex (S1Left) of monkey A. The array consisted of sixteen electrodes inserted about 1 mm into the cortex. The average fraction of highly correlated sites across suprathreshold nLFPs (i.e., R≥0.8, shaded area in Figure 1E) increased sigmoidally with nLFP amplitude beyond ∼1 SD (sigmoidal fit, R>0.99) and was significantly different from all controls (red, green; p<10−4 all cases). For further information on controls, see Figure 1 and main text. (B) The distribution of temporal differences between highly correlated nLFPs (≤−3 SD; R≥0.8; best-match comparison) centers around 0 as found for other cortical areas in vivo and in vitro (cf., Figure 2B). The distribution is similar for peak-to-peak time differences (red). (0.38 MB TIF) [file pbio.1000278.s002.tif]

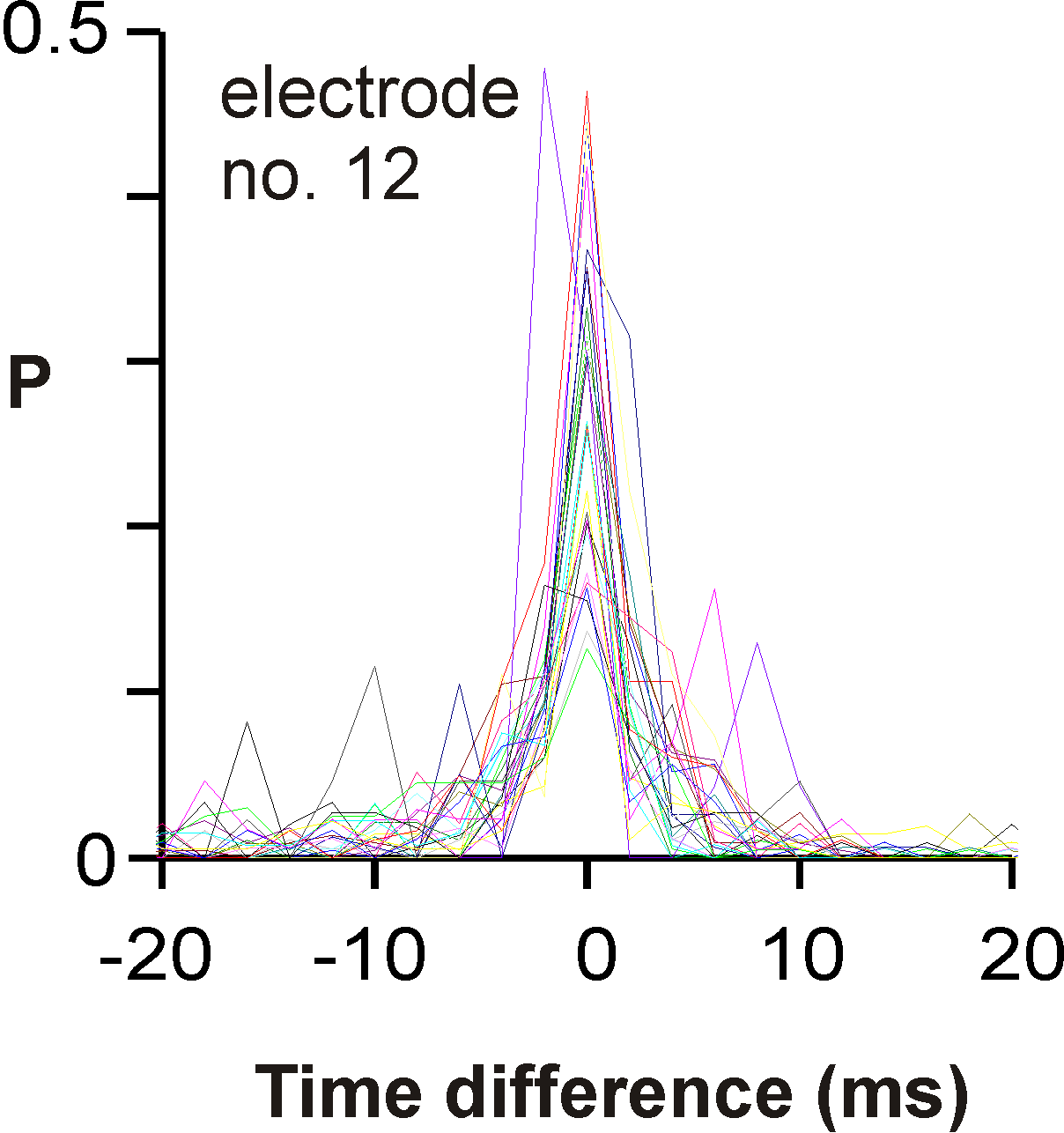

Supplement: Figure S3 — Distribution of time differences was similar between all electrode pairs. The distributions of time differences between coherence potentials on each pair of electrodes on the array are similar to each other. Shown here are the comparisons between one electrode and every other electrode on the array for the M1Left region of monkey A. The lack of any characteristic differences between any electrodes suggests that the time differences arise due to functional parameters rather than structural or anatomical differences due to electrode positioning. (0.18 MB TIF) [file pbio.1000278.s003.tif]

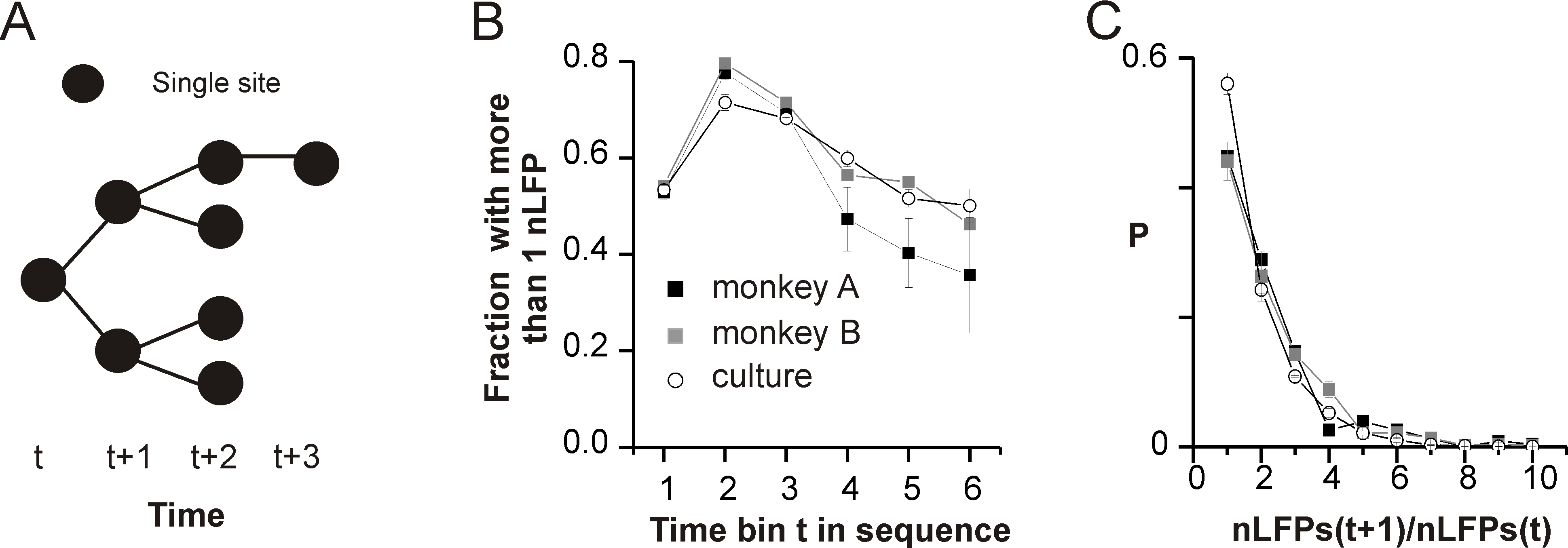

Supplement: Figure S4 — Coherence potential propagation is consistent with a cascade model. (A) A cascade model of propagation where activity originates from a single site and propagates in a one-to-many fashion for successive time bins. (B) Consistent with a one-to-many process, the first time bin had a much lower likelihood of having multiple nLFPs compared to the next two time bins. Graph shows fraction of cases in each successive time bin where there was more than one correlated nLFP. Analysis uses only nLFP sequences extending to ten or more sites (M1left, monkey A; average over four arrays in monkey B; average over n = 6 cultures). (C) The probability of propagating to n sites, approximated by the number of nLFPs in the (n+1)th time bin relative to the nth time bin, decreased according to an exponential function (0.83e(−n/1.7)M, R 2 = 0.98 in vivo and 1.2e(−n/1.2), R 2 = 0.99 in vitro). Same legend as in B. (0.34 MB TIF) [file pbio.1000278.s004.tif]

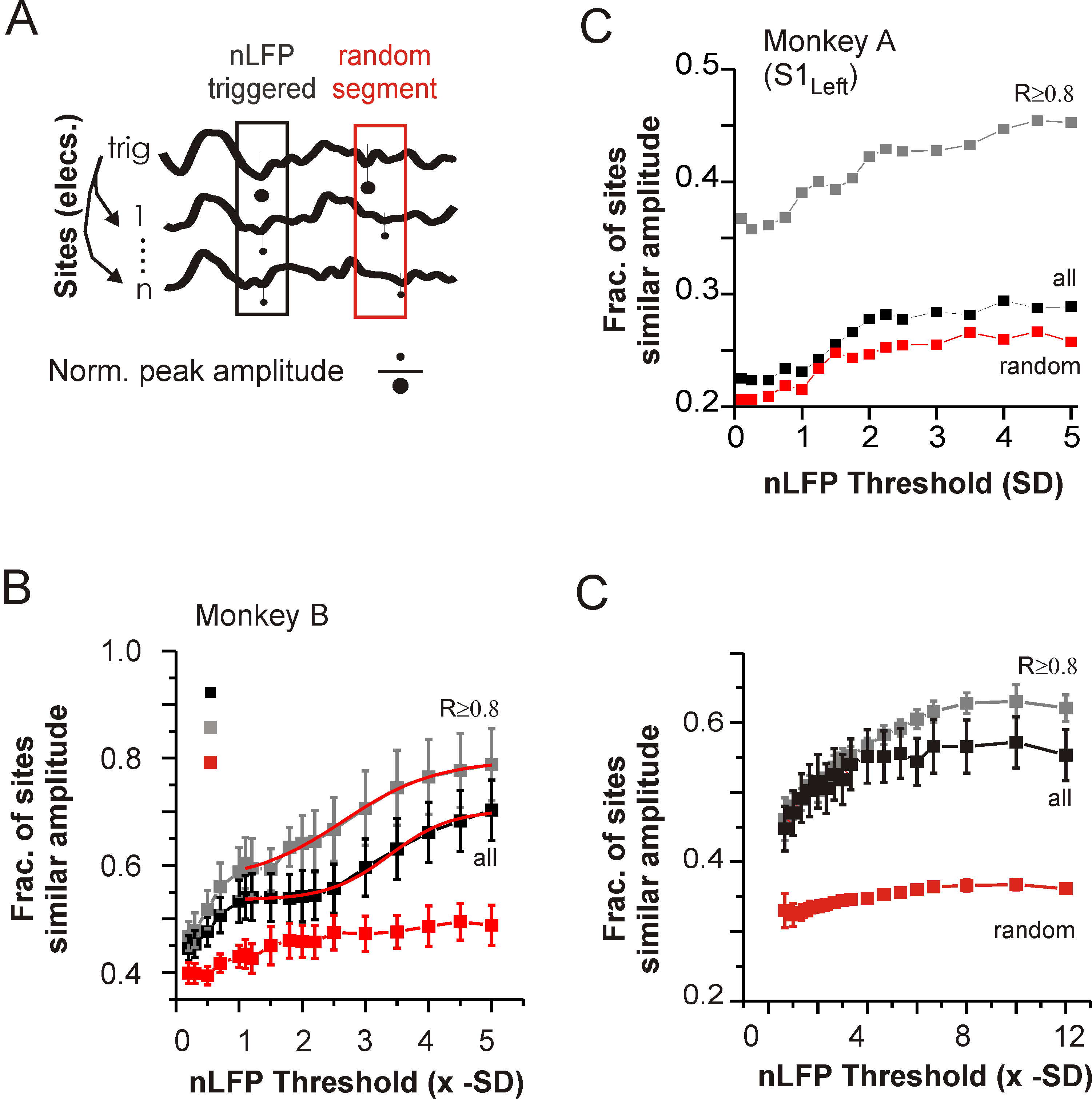

Supplement: Figure S5 — Correlated periods are similar in peak amplitude. (A) Normalized peak amplitude comparisons as in Figure 2F–2H. (B) Fraction of sites with peak amplitude within ±50% of the nLFP trigger amplitude for the somatosensory array (S1Left) in monkey A increased non-linearly as a function of trigger amplitude and was significantly greater for correlated waveforms alone. (C) Similar result as in B for monkey B (average over four arrays). (D) Similar result as in B for cultures (average over n = 6 cultures). (0.53 MB TIF) [file pbio.1000278.s005.tif]

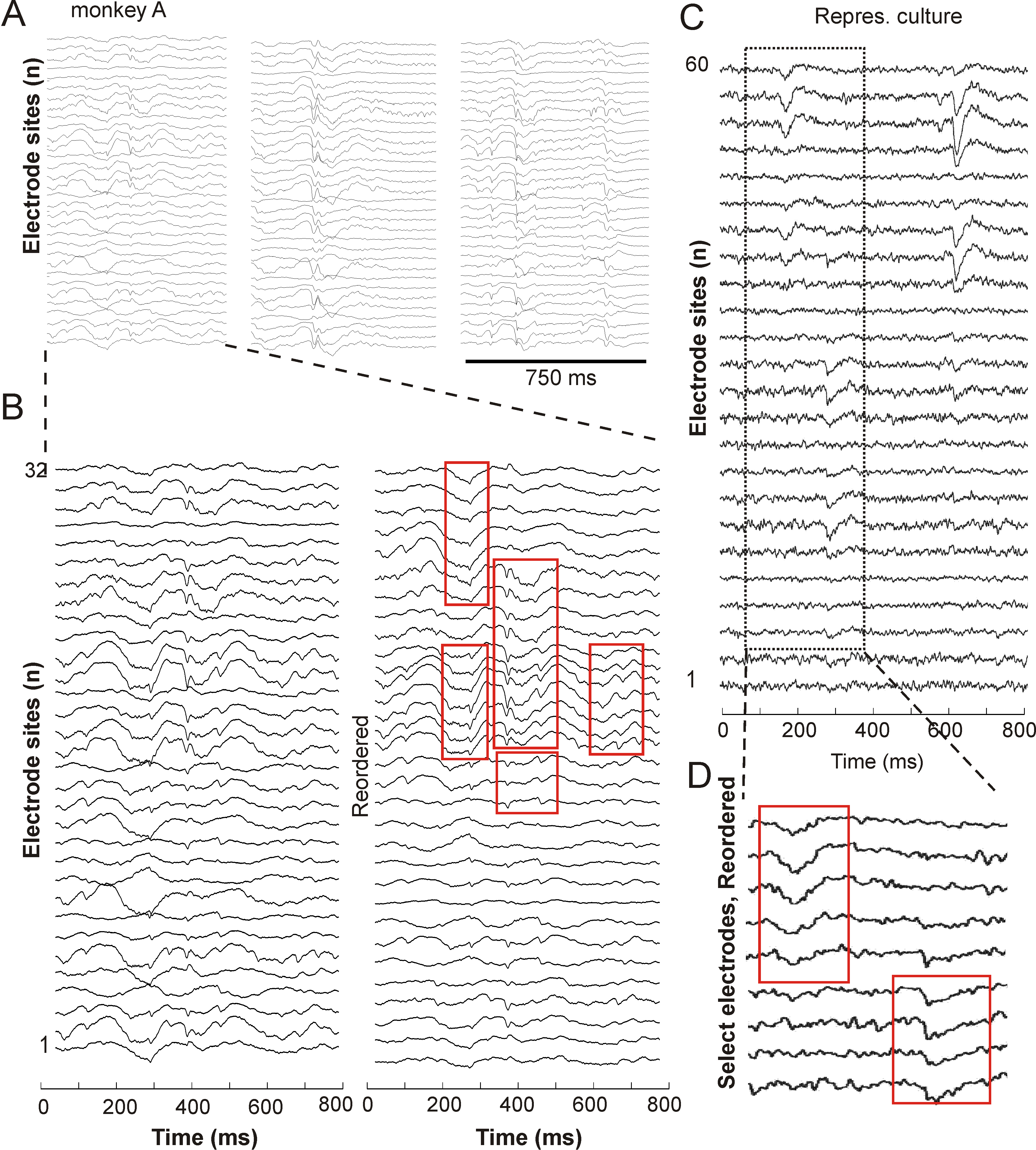

Supplement: Figure S6 — Raw traces of periods encompassing one or more coherence potentials. (A) Raw traces of three 750 ms periods encompassing one or more coherence potentials from all thirty-two electrodes in M1Left in monkey A. (B) Blow up of the first 750 ms period (left). Same 750 ms period after rearranging the electrode order (right). Rearranged panel shows five distinct coherence potential sequences (red boxes) spanning different subsets of electrodes, providing demonstration of successive within and between sequence comparisons. (C) Raw traces of all 60 electrodes corresponding to one 750 ms period from one culture encompassing one or more coherence potentials. (D) Blow up of signal during period corresponding to box in C for select electrodes, with order rearranged. (1.54 MB TIF) [file pbio.1000278.s006.tif]

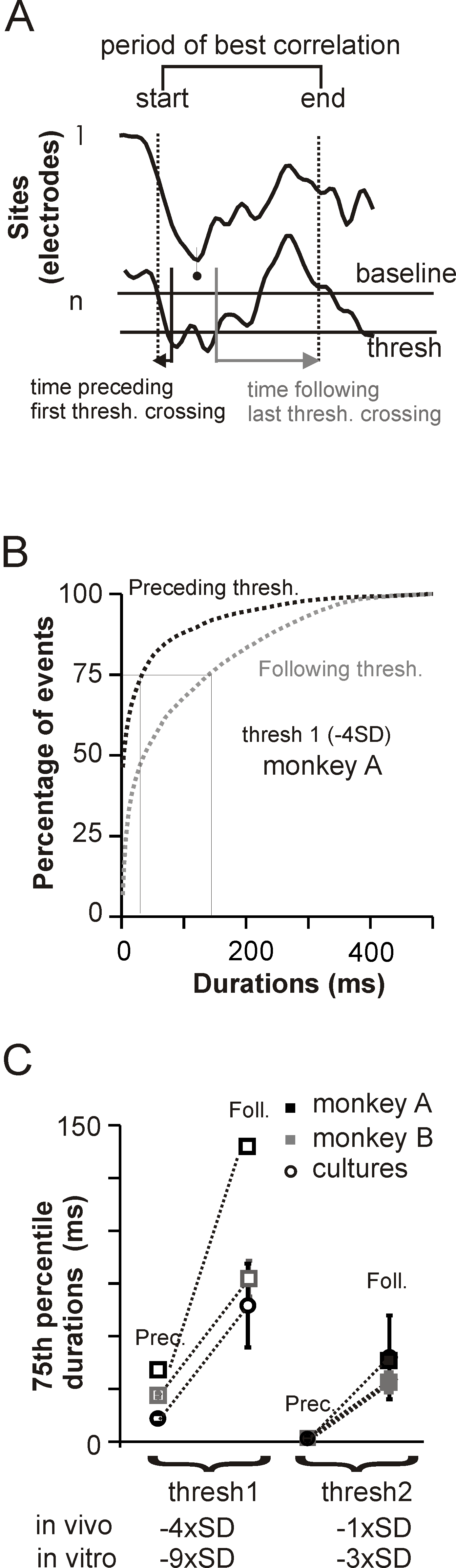

Supplement: Figure S7 — Correlated periods are initiated at large amplitude crossings. (A) The onset of maximal waveform correlation preceding the first threshold crossing (preceding thresh) and following the last threshold crossing (following thresh) was identified by progressively increasing the window of comparison in each direction. Because coherence potentials almost always included both a positive and negative excursion in a yet undetermined order, we considered threshold crossings in both directions. As our first amplitude threshold (thresh 1) we chose the first value that gave rise to the maximal fraction of correlated sites (cf., Figure 1C; ±4 SD in vivo and ±9 SD in vitro). (B) Distributions of durations (identified as in A) correlated with R≥0.8 preceding the first crossing of threshold in either the positive or negative direction (thresh 1: ±4 SD in vivo, ±12 SD in vitro; thresh 2: ±1 SD in vivo, ±3 SD in vitro). In monkey A, while ∼50% began at or after the first crossing of 4 SD, only ∼6% ended at or before the last threshold crossing. The pattern was similar for monkey B (22±2 ms preceding, 76±13 ms following) and in vitro (11±4 preceding, 65±41 following) indicating that while coherence potentials were initiated shortly before a large amplitude crossing, they could continue well beyond the last crossing. Of these, ∼60% in vivo and ∼72%±3% in vitro were initiated by negative amplitude crossings while the remaining fraction was initiated by positive amplitude crossings, indicating the presence of an inverse pattern, i.e. negative-positive and positive-negative. This lack of clear directionality likely reflects the positioning of neuronal activity relative to the electrodes and therefore whether the electrode measures the sink or source of the activity but may also reflect differences in the arrival of excitatory and inhibitory input or relationships between spike occurrence and the associated synaptic currents. (C) 75th percentile values of durations from distributions constr [file pbio.1000278.s007.tif]

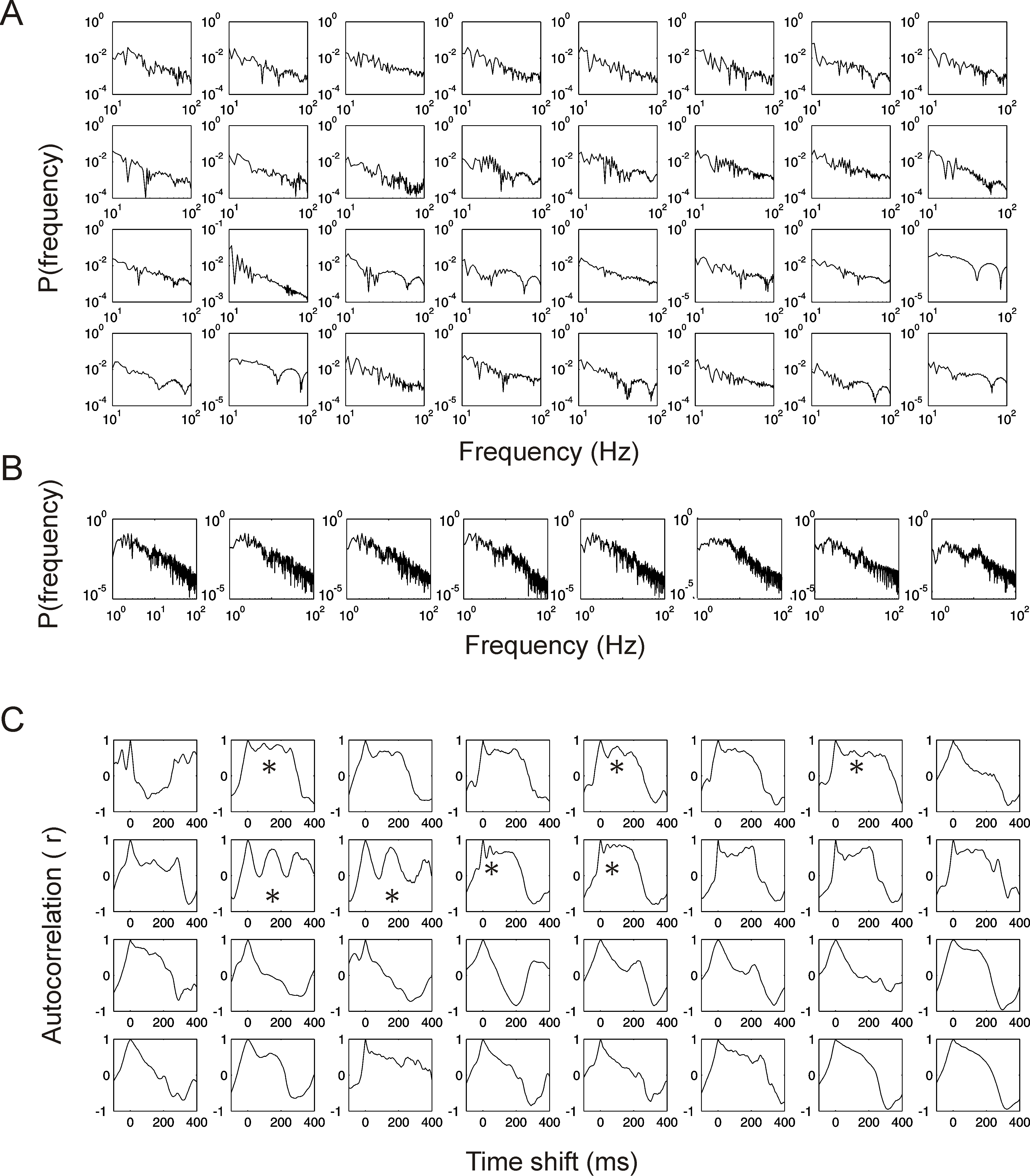

Supplement: Figure S8 — Power spectrums and autocorrelations for individual coherence potential waveforms. (A) Power spectrums (in log-log coordinates, shown from 10–100 Hz) calculated as the FFT of the autocorrelation for forty distinct, randomly selected coherence potentials ≥200 ms in duration. The power spectrum is broad and generally decays without any dominant frequency. (Note that this method is different from that shown in Figure 4 but is in agreement with the findings where the FFT was applied to the signal itself.) (B) Examples of power spectrums (in log-log coordinates, shown for 1–100 Hz) for periods extending ±4 s from the nLFP peak do not show any clear characteristic low frequency. (C) Autocorrelations of the coherence potentials in A, obtained by taking the nLFP period and calculating its correlation to time shifted segments of equivalent duration from the same signal. For all cases, autocorrelations were calculated up to 50 ms preceding the peak of the nLFP and 350 ms after the peak using corresponding time segments around the coherence potentials. Autocorrelations were highly variable with a small subset revealing an oscillatory component (marked with a *). (1.31 MB TIF) [file pbio.1000278.s008.tif]

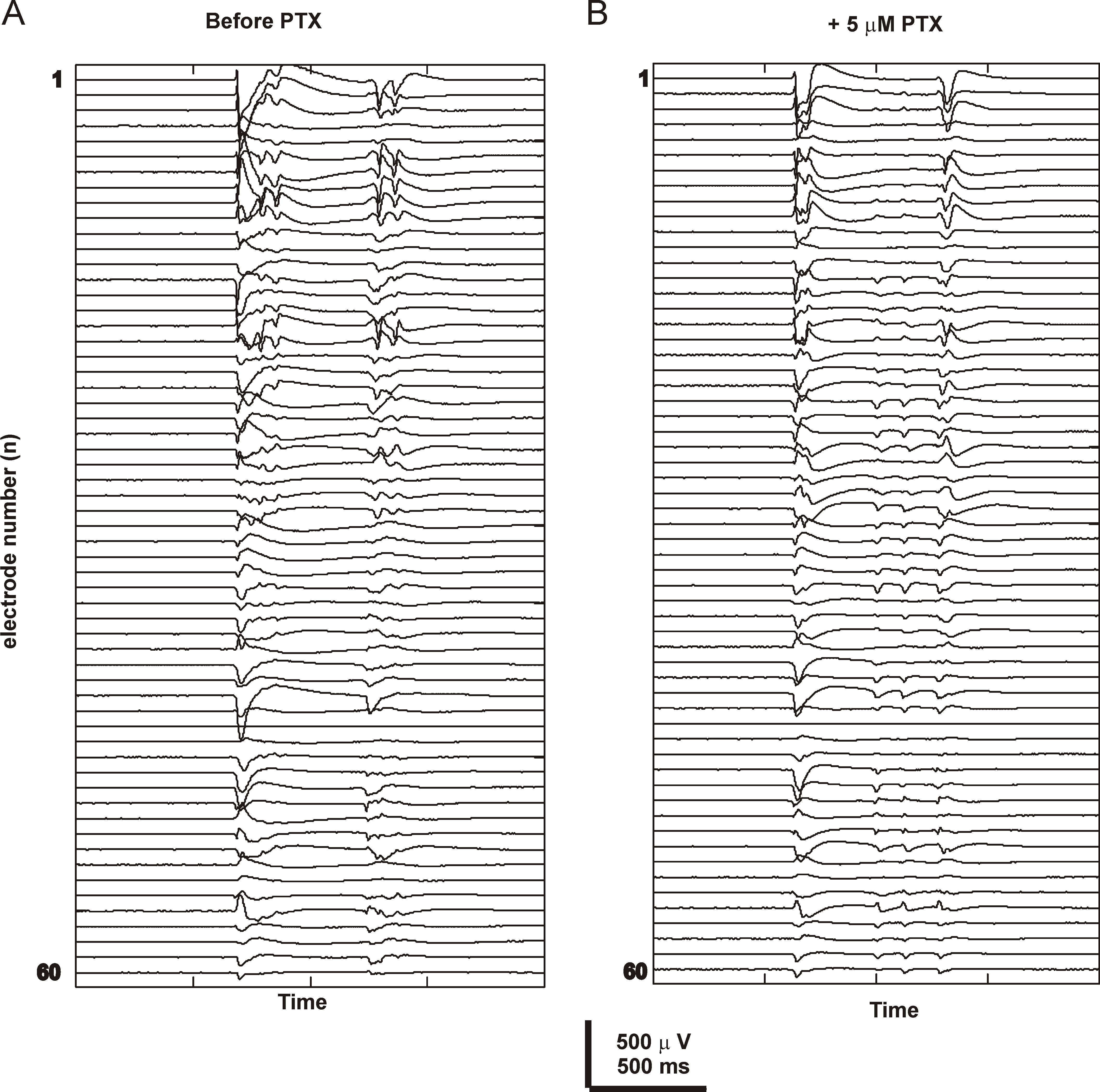

Supplement: Figure S9 — Raw traces of in vitro recordings before and after application of 5 µm picrotoxin (PTX). (A) Period of spontaneous neuronal activity in a single cortex culture. (B) Spontaneous activity in the presence of 5 µM of the GABAA-receptor antagonist PTX, which reduces fast synaptic inhibition (same culture). (1.06 MB TIF) [file pbio.1000278.s009.tif]

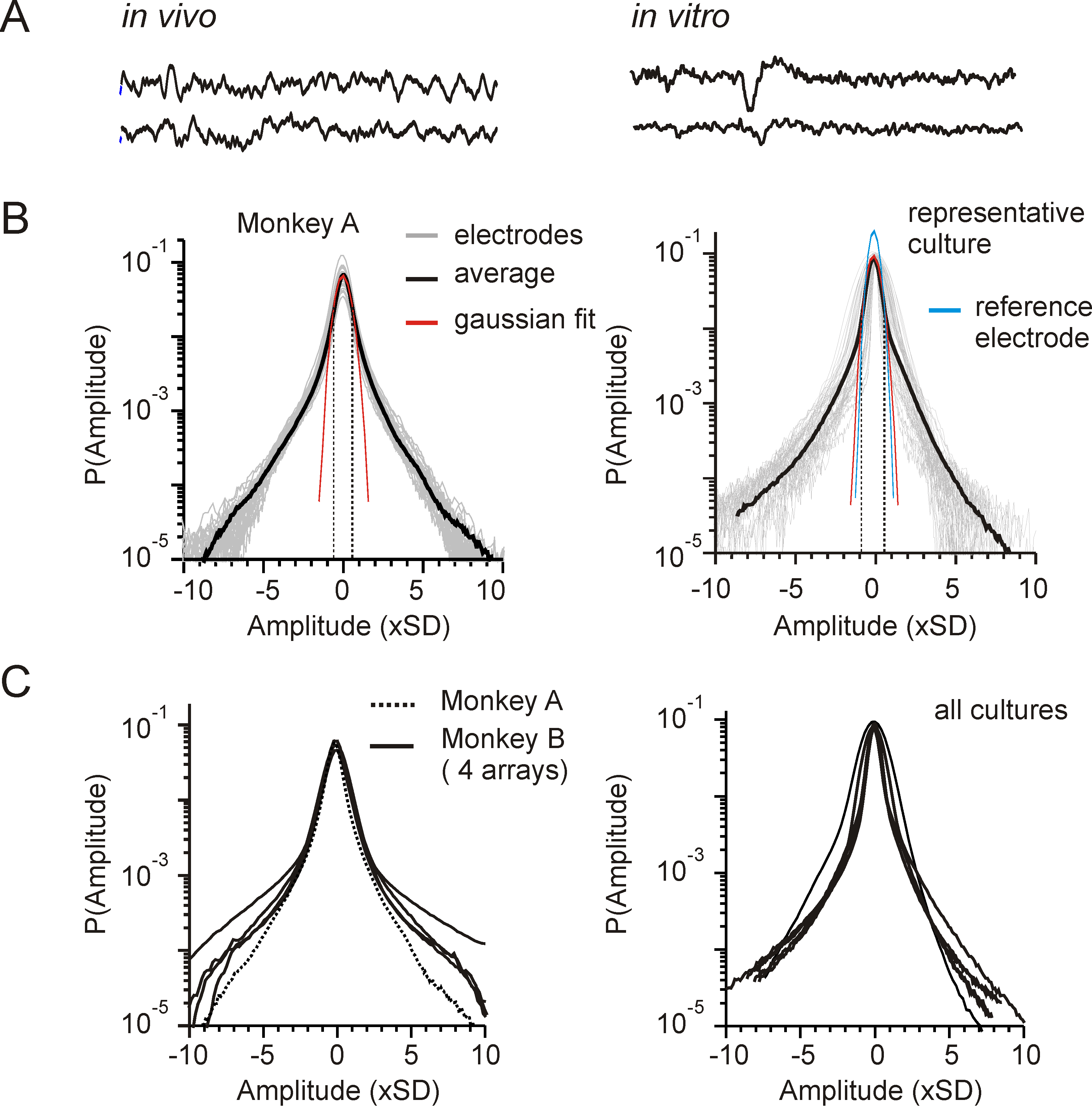

Supplement: Fiugre S10 — The LFP signal deviates from Gaussian noise at large amplitudes. (A) Two second long traces of local field potential (LFP) activity recorded from two electrodes of one in vivo array (left) and one in vitro array (right). (B) Histograms of the LFP amplitudes at each electrode (gray) and average (black). Amplitude scale is in multiples of standard deviation (×SD) of the average activity for a representative array in vivo (left) and in vitro (right). Ordinate axis is shown in log units to emphasize tails which deviate significantly from the best Gaussian fit (red). Gaussian fit is closely mirrored by instrument noise measured at the reference electrode (blue, in vitro only). The deviation of amplitudes from the Gaussian fit occurred roughly in the range of 0.75 SD (in vivo) to 1.5 SD (in vitro), indicating that beyond these values the behavior of the signal was likely to be highly distinct from noise. (C) Average LFP amplitude histogram for each of the four arrays in monkey B (left) and six arrays in vitro (right) show similar patterns. (0.76 MB TIF) [file pbio.1000278.s010.tif]

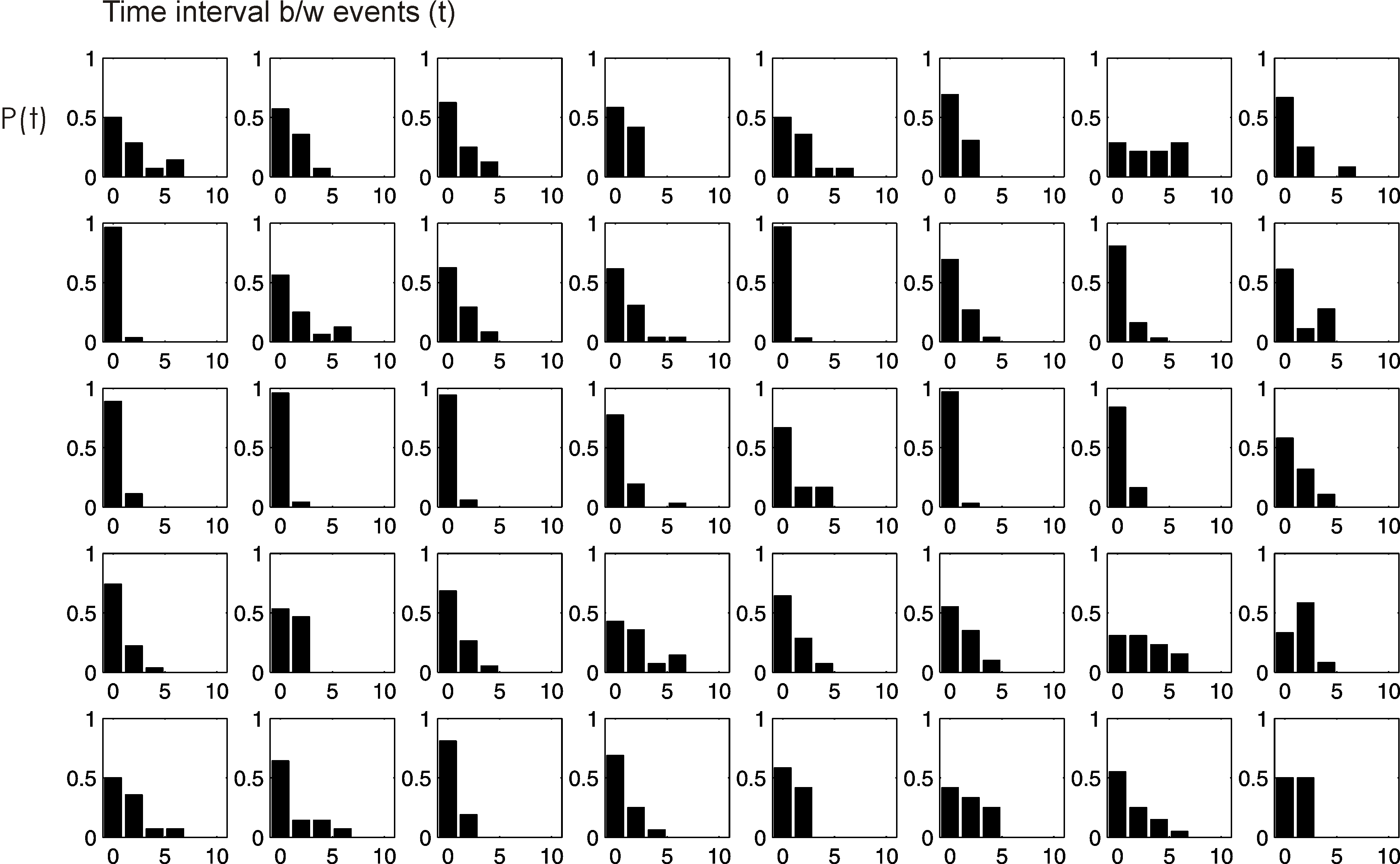

Supplement: Figure S11 — Intervals between successive nLFPs of individual coherence potentials. Distributions of intervals between successive highly correlated nLFPs (i.e., coherence potentials). Shown are forty such coherence potentials spanning >10 electrode sites (individual boxes). Wide range of intervals or delays indicates no characteristic time scale. This result is inconsistent with coherence potentials arising on different cycles of an oscillation. (0.68 MB TIF) [file pbio.1000278.s011.tif]

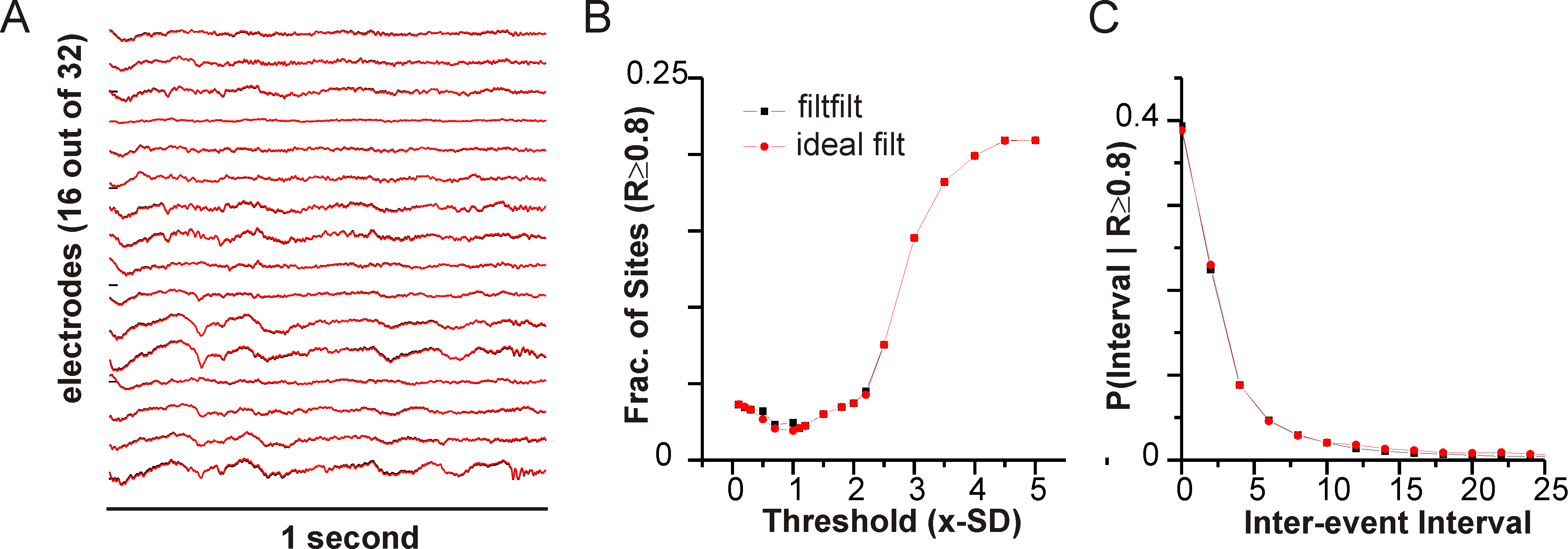

Supplement: Figure S12 — Comparison of forward and forward-backward filters for monkey A. (A) Segment of recording at sixteen of thirty-two electrodes from monkey A (M1Left) filtered with a forward filter (“idealfilt” in MATLAB) and a phase neutral forward-backward filter (“filtfilt” in MATLAB) shows high similarity. (B) Sigmoidal transition to a regime of high spatial coherence (time-aligned analysis as in Figure 1F for M1Left; monkey A) does not depend on the filter method. (C) Corresponding probability density of time intervals between successive, correlated sites (see Figure 2B main text) does not depend on the filter method. (0.39 MB TIF) [file pbio.1000278.s012.tif]
